# Constraint-Based Model of *Shewanella oneidensis* MR-1 Metabolism: A Tool for Data Analysis and Hypothesis Generation

**DOI:** 10.1371/journal.pcbi.1000822

**Published:** 2010-06-24

**Authors:** Grigoriy E. Pinchuk, Eric A. Hill, Oleg V. Geydebrekht, Jessica De Ingeniis, Xiaolin Zhang, Andrei Osterman, James H. Scott, Samantha B. Reed, Margaret F. Romine, Allan E. Konopka, Alexander S. Beliaev, Jim K. Fredrickson, Jennifer L. Reed

**Affiliations:** 1 Biological Sciences Division, Pacific Northwest National Laboratory, Richland, Washington, United States of America; 2 Burnham Institute for Medical Research, La Jolla, California, United States of America; 3 Department of Chemical and Biological Engineering, University of Wisconsin-Madison, Madison, Wisconsin, United States of America; 4 Department of Earth Sciences, Dartmouth College, Hanover, New Hampshire, United States of America; Medical College of Wisconsin, United States of America

## Abstract

Shewanellae are gram-negative facultatively anaerobic metal-reducing bacteria commonly found in chemically (i.e., redox) stratified environments. Occupying such niches requires the ability to rapidly acclimate to changes in electron donor/acceptor type and availability; hence, the ability to compete and thrive in such environments must ultimately be reflected in the organization and utilization of electron transfer networks, as well as central and peripheral carbon metabolism. To understand how *Shewanella oneidensis* MR-1 utilizes its resources, the metabolic network was reconstructed. The resulting network consists of 774 reactions, 783 genes, and 634 unique metabolites and contains biosynthesis pathways for all cell constituents. Using constraint-based modeling, we investigated aerobic growth of *S. oneidensis* MR-1 on numerous carbon sources. To achieve this, we (i) used experimental data to formulate a biomass equation and estimate cellular ATP requirements, (ii) developed an approach to identify cycles (such as futile cycles and circulations), (iii) classified how reaction usage affects cellular growth, (iv) predicted cellular biomass yields on different carbon sources and compared model predictions to experimental measurements, and (v) used experimental results to refine metabolic fluxes for growth on lactate. The results revealed that aerobic lactate-grown cells of *S. oneidensis* MR-1 used less efficient enzymes to couple electron transport to proton motive force generation, and possibly operated at least one futile cycle involving malic enzymes. Several examples are provided whereby model predictions were validated by experimental data, in particular the role of serine hydroxymethyltransferase and glycine cleavage system in the metabolism of one-carbon units, and growth on different sources of carbon and energy. This work illustrates how integration of computational and experimental efforts facilitates the understanding of microbial metabolism at a systems level.

## Author Summary

The role of members of the genus *Shewanella* in global carbon and nutrient cycles is implicated based on their wide distribution and their ability to link organic matter oxidation to reduction of many organic and inorganic electron acceptors both natural and artificial. These bacteria are also important in bioremediation efforts and other developing fields, including energy generating biocatalysis and the production of useful carbon containing compounds. In this study we applied a suite of systems-biology tools, including computational and high-throughput experimental approaches, to develop a predictive understanding of *S. oneidensis* MR-1 metabolism. We built a metabolic model and used it to analyze experimental data and to predict cellular phenotypes. Predicted cellular biomass yields on different carbon sources under aerobiosis were compared to experimental measurements, and experimental results were used to refine metabolic fluxes for growth on lactate. Computational and experimental analysis of *S. oneidensis* MR-1 metabolism revealed some of the reasons for a significant difference between experimental and predicted aerobic growth efficiency on lactate. The developed model provides a platform for a systematic assessment of *Shewanella* metabolism, which may be used for redesigning metabolic networks for chemical production.

## Introduction

*Shewanella* are common organoheterotrophic organisms in both marine and fresh water environments, particularly in those receiving high inputs of organic matter and where redox conditions fluctuate in space and time. *Shewanella oneidensis* MR-1 is a dissimilatory manganese-reducing bacterium isolated from Lake Oneida in upstate New York [1] and is among the best studied members of this genus. It grows well on three-carbon substrates such as lactate and pyruvate, but can also use a range of other compounds as sole carbon and energy sources, including protein and DNA [2], [3] and *N*-acetylglucosamine [4]. *Shewanella* is particularly well-adapted to redox interface environments [5] where electron donor (carbon substrate) is abundant but electron acceptors can be limiting and variable over short distances. Many members of this genus can utilize a wide range of electron acceptors, including O_2_, fumarate, nitrate, nitrite, sulfite, tetrathionate, thiosulfate, TMAO, DMSO, Fe(III), and Mn(VI). Given its propensity to transfer electrons to extracellular substrates, *Shewanella* has been of interest for use in microbial fuel cells [6]–[8]. Because of its ability to reduce metals and radionuclides it has also been used as a model organism for investigating redox transformations of environmental contaminants such as uranium [9] and technetium [10].

*Shewanella* are obligately respiring bacteria; however, *S. oneidensis* MR-1 has recently been shown to survive by fermenting pyruvate [11]. These bacteria use a limited range of substrates for growth by anaerobic respiration (lactate, pyruvate, and DNA [1], [2], [12]), whereas aerobic growth is supported by a larger range of carbon containing compounds. Of particular interest is the inability of *Shewanella*, unlike *Geobacter* [13] and *Anaeromxyobacter* [14], to grow by coupling acetate oxidation to reduction of electron acceptors other than O_2_ [12]. Moreover, growth of *Shewanella* on lactate under anaerobic conditions is accompanied by acetate accumulation [12]. Elucidation of the mechanisms underlying these and other metabolic traits is important to gain a deeper understanding of the roles of *Shewanella* in catalyzing important environmental processes, as well as to assess and improve their potential for biotechnological applications. Genome-scale metabolic models allow for a systematic assessment of the metabolic capabilities of an organism. These models can be used to analyze and predict the metabolic behavior of microorganisms under different environmental conditions and in response to genetic alterations [15]. As such, these models have been used in metabolic engineering for designing industrial production strains *in silico* [16]–[18].

In this work, we developed a metabolic reconstruction for *S. oneidensis* MR-1 and analyzed a genome-scale constraint-based model of MR-1 metabolism to interrogate the organism's metabolic behavior and capabilities under aerobic conditions and to estimate the growth- and non-growth rate dependent ATP requirements. We made detailed biomass composition measurements for macromolecules (DNA, RNA, lipids, protein, and carbohydrates) and amino acids. These measurements were used to construct a biomass production reaction that was included in the metabolic model iSO783. The resulting model was used to identify futile cycles, predict biomass yields on alternative carbon and energy sources, and refine intracellular flux distributions for lactate-limited growth by integrating experimental data. We also illustrate how models can be used to improve understanding of microbial metabolism by incorporating experimental data to refine metabolic pathways reconstructed from a genome annotation as well as to predict how cells will behave in response to genetic or environmental perturbations.

## Results

### *S. oneidensis* MR-1 Biomass Composition and Metabolic Reconstruction

The biomass composition was measured experimentally for *S. oneidensis* MR-1 grown in a lactate-limited chemostat under aerobic conditions with a dilution rate (D) = 0.095 h^−1^ and contained 52.8% protein, 17.5% lipids, 7.7% carbohydrates, 9% RNA, and 5% DNA on a g/g AFDW (ash-free dry weight) basis. The remaining biomass constituents (peptidoglycan-2.5%, lipopolysaccharides-3.4%, and soluble intracellular pools-3.1%) were assumed to have similar abundances as in *E. coli* [19]. The comparison of AFDW values to the sum of all cellular components measured in complete liquid culture revealed that the difference between them never exceeded 3%, which is in the error range for the methods used. This observation indicates that *S. oneidensis* MR-1 did not excrete a significant amount of polymers under these growth conditions and that our approximations for un-measured biomass components were reasonable. The nucleotide composition of RNA and DNA were estimated based on the GC content of MR-1, and the amino acid composition of the proteins and the cellular lipid composition were based on experimental measurements. Together this information was used to formulate a biomass equation for the network (see Table S1 for complete biomass details), which was used as an objective function to predict intracellular flux distributions and growth rates. Intracellular fluxes are expected to be more sensitive to changes in biomass composition (due to incorrect estimates of unmeasured biomass components or resulting from different growth conditions) than calculated biomass yields and energy requirements [20], [21].

The *S. oneidensis* MR-1 metabolic network was reconstructed using an automated procedure followed by manual curation (see Materials and Methods for details). Manual curation was carried out to verify the reactions included in the automodel were correct and to generate organism-specific biosynthesis pathways (such as phospholipids, lipopolysaccharides, and quinones). The result is a final reconstruction that includes 774 reactions, 783 genes, and 634 unique metabolites (see Tables S2 and S3 for network details) and contains biosynthesis pathways for all amino acids, nucleotides, lipids, and for a number of cofactors (NAD, NADP, FAD, CoA, acyl carrier protein, folate, quinones, pyridoxal 5-phosphate, and hemes). Particular attention was given to electron transport where the primary literature (see Table S2 for references) and *S. oneidensis* MR-1 annotated genome were used to manually reconstruct the pathways leading to the reduction of various electron acceptors

An important issue in the reconstruction of metabolism is the correct accounting of ATP production during substrate oxidation. Reconstructing the electron transport pathways can be challenging since energy conservation during respiration varies as a function of organism and growth condition [22], [23]. Three terminal oxidases, which use O_2_ as the electron acceptor, were included in the metabolic network: two cytochrome c oxidases (Cco, SO2361–2364; Cox, SO4606–4607, SO4609) and one cytochrome d ubiquinol oxidase (Cyd, SO3285–3286). The use of either Cco or Cox, in combination with ubiquinol-cytochrome c reductase (Pet, SO0608–0610), results in the translocation of 6H^+^/2e^−^ across the cytoplasmic membrane as electrons move from ubiquinol to O_2_ [24]. However, the use of Cyd only results in the translocation of 2H^+^/2e^−^ as electrons are transferred from ubiquinol to O_2_. Previous proton translocation measurements for *S. oneidensis* MR-1 with oxygen as the electron acceptor found that a maximum of 2.8H^+^/2e^−^ are translocated when cells are grown aerobically [25]. This measurement implies that the flux through Cyd is four times higher than the combined flux through Pet-Cco and Pet-Cox; therefore, all aerobic simulations reported in the results sections below were performed using this flux ratio constraint (see discussion for how the assumed H^+^/e^−^ affects results).

### Estimation of ATP Requirements

**Figure 1:**
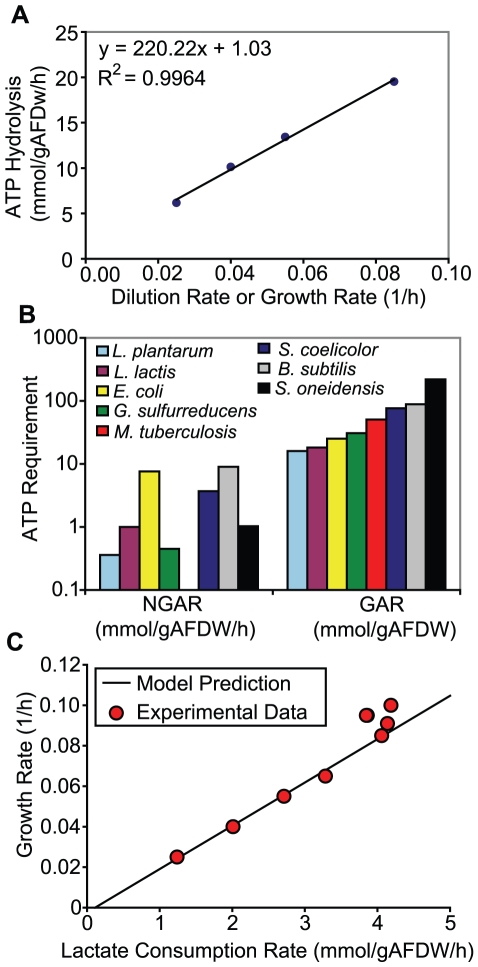
ATP requirements for maintenance and growth. Panel A shows the model estimated maximum ATP hydrolysis rates needed to match experimentally measured lactate consumption rates and cellular growth rates at four different dilution rates (D = 0.025. 0.04, 0.055, 0.085 h^−1^). The slope and intercept represent the growth- and non-growth rate dependent ATP requirements, GAR and NGAR, respectively. Panel B shows ATP requirements for various microbes that have been reported in the literature [26]–[32]. The reported GAR values for other microbes were adjusted to remove ATP used for protein polymerization (4 ATP/peptide bond) since ATP used for protein synthesis is accounted for separately in the *S. oneidensis* MR-1 model and is not part of the MR-1 GAR value. Panel C compares model estimates of maximum growth rates (solid line) at different lactate consumption rates (using ATP requirements as reported in panel A) with experimental data. Additional data points were included that were not used in the estimation of the ATP requirements.

To estimate *S. oneidensis* MR-1 ATP requirements, the lactate consumption rate was measured at different dilution rates in a chemostat. Analysis of cultural liquid revealed no detectable amounts of organic acids and that residual lactate concentrations were below 0.1 mM (the detection limit of the quantification method used); therefore all the lactate added to the medium (18mM) was consumed by the bacteria. Using the lactate consumption rate as a model constraint, we calculated the maximal amount of ATP that could be hydrolyzed while still maintaining the measured growth rate. A linear relationship was found between dilution rates (D) and maximum ATP hydrolysis (Figure 1A), where the slope represents the growth rate-dependent ATP requirements (GAR), and the intercept the non-growth rate dependent ATP requirement (NGAR) [26]. The maximal rate of ATP hydrolysis then represents GAR multiplied by the cellular growth rate plus NGAR. In our model GAR accounts for the energy expenditure on unknown processes that may include protein and mRNA turnover or repair, proton leakage, and maintenance of membrane integrity, but does not account for ATP spent on polymerization reactions, as this is directly accounted for in the macromolecular synthesis reactions included in the metabolic network.

From the experimental growth and lactate consumption rates, the model estimated NGAR to be 1.03 mmol ATP/(g AFDW•h), and GAR to be 220.22 mmol ATP/g AFDW, when the transfer of electrons from ubiquinol to O_2_ has a proton translocation efficiency of 2.8H^+^/2e^−^. This GAR is significantly higher than values reported for other microorganisms (Figure 1B) [26]–[32]. Using the estimated ATP requirements as parameters in the model, we calculated the maximum growth rate as a function of lactate consumption rate and compared it to experimental measurements (Figure 1C), which included additional growth rates not used in the estimation of the ATP requirements. Interestingly, at growth rates above 0.085 h^−1^, *S. oneidensis* MR-1 was able to grow more efficiently than predicted by the model (experimental biomass yield was higher than predicted yield), while at growth rates less than or equal to 0.085 h^−1^, the model-estimated biomass yields were in good agreement with experimental values. This may imply that at lower dilution rates the cells could be using metabolic pathways that reduce energetic efficiency.

### Futile Cycles & Suboptimal Pathways

Futile cycling occurs when opposing reactions catalyzed by different enzymes take place simultaneously, resulting in a dissipation of energy. Given the high apparent GAR value for *S. oneidensis* MR-1 compared to other evaluated bacteria (Figure 1B), we hypothesized that energy-dissipating futile cycles may operate in *S. oneidensis* MR-1 under aerobic conditions. Since the bacteria are not exposed to high O_2_ concentrations in their environment they may not be adapted to O_2_ rich environments, but rather to growth in suboxic and anoxic environments enriched with other electron acceptors besides O_2_ [5]. To assess this issue, we developed a new optimization-based approach to identify ATP-dependent futile cycles in the network (see Materials and Methods). The approach can also be used to identify cycles with no net transformations (e.g. circulations [26]) or cycles where the net reaction is a transhydrogenase activity. The smallest one hundred and thirty futile cycles (i.e. those containing the fewest number of reactions, which are likely to be more biologically realistic) in the iSO783 metabolic network were found computationally (listed in Table S4), each containing between 2 and 11 reactions.

**Figure 2:**
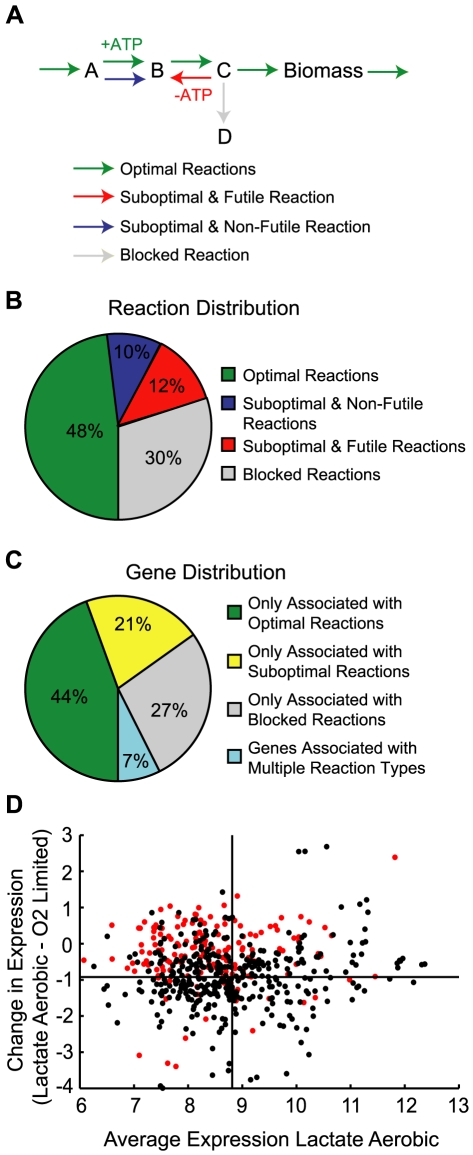
Classification of reactions and genes for lactate-limited aerobic growth. Panel A illustrates the classification of reactions based on how fluxes through the reactions affect biomass production. Optimal reactions are ones that can be used to achieve maximal growth rates, these are the most efficient pathways. Suboptimal reactions are ones where non-zero fluxes force a reduction in maximal growth rate. These reactions can be further classified as futile (meaning they participate in futile cycles) or non-futile (they do not participate in futile cycles; these are often less energetically efficient pathways). Blocked reactions are ones that can not carry any flux due to the imposed constraints, so all solutions, optimal and suboptimal, will have zero flux through the reactions. The classification of the reactions is highly dependent on the growth condition. Panel B shows the distribution of reactions in iSO783 for lactate-limited aerobic growth. Panel C shows the distribution of genes in the model based on their association to the classified reactions. For example, if a gene is only associated with optimal reactions then it is classified as optimal, but if it is associated with an optimal reaction and a futile cycle reaction then it is classified as associated with multiple reactions. Panel D shows the expression (reported as RMA, Robust Multichip Average) in lactate limited aerobic conditions versus the change in expression from aerobic conditions to oxygen-limited for genes associated with optimal reactions (black, 387 genes) and with suboptimal reactions (red, 181 genes). The black horizontal and vertical lines show the mean expression and mean expression changes for the optimal set of genes. Only 39 genes associated with suboptimal reactions fall in the upper right quadrant. Gene expression data was obtained from the M3D database [35].

A number of the reactions participating in these futile cycles are necessary for the maximum production of biomass, and it is the flux through their reaction partners that dissipates ATP and reduces biomass production (Figure 2A). To identify which portion of a futile cycle was needed for optimal biomass production and which portion caused futile cycling, flux variability analysis (FVA) [33] was used to determine fluxes through reactions during optimal and suboptimal biomass production and ATP dissipation (see Materials and Methods). These FVA results allowed us to classify all reactions in the metabolic network as being:

- optimal— meaning a non-zero flux through the reaction can lead to maximum biomass production
- suboptimal and futile— a non-zero flux through the reaction reduces biomass production and the reaction is part of a futile cycle
- suboptimal and non-futile— a non-zero flux through the reaction reduces biomass production but the reaction is not part of a futile cycle; for example, a less energetically efficient alternative pathway
- blocked— no flux through the reaction is possible at steady state

While the enumerated futile cycles themselves are independent of growth condition, the classification of the fluxes by FVA depends on the growth condition used in the simulation. For lactate-limited aerobic growth, the distribution of reactions (Figure 2B) and their associated genes (Figure 2C) in the iSO783 network into the different categories was determined. These reaction distributions are similar to those found for *E. coli* during optimal growth in aerobic minimal media [34].

Once reactions and genes are classified, various types of datasets (e.g., gene expression, proteomic, metabolomic) can be used to identify what suboptimal reactions are potentially used that might explain the high GAR values. We evaluated available gene expression data for lactate aerobic and O_2_-limited growth obtained from the M3D database [35] and focused our analysis on the 181 genes that were associated with suboptimal reactions (either futile or non-futile) and not optimal reactions. Thirty-nine of these 181 genes had both: (i) higher expression under aerobic conditions than the average expression of 387 genes associated with optimal reactions; and (ii) higher change in expression between aerobic and O_2_-limited conditions, as compared to the average change in expression for genes associated with optimal reactions (i.e., 39 red dots fall within upper right quadrant of Figure 2D).

Based on this gene expression analysis, it appears that some futile cycles may be operational during aerobic growth on lactate. These include pyruvate kinase and phosphoenolypyruvate synthase and fatty acid synthesis and degradation. Phosphoenolpyruvate carboxylase was also picked up in our analysis of the expression data, and it participates in futile cycles with malic enzymes. Experimental assessment revealed that when either of the two malic enzymes (*SO3855* or *SO4118*) was deleted, the cells showed a ∼25% increase in biomass yield (indicated by higher final ODs) over the wild-type strain in lactate aerobic batch cultures (Figure S1A), indicating that futile cycles involving malic enzyme may reduce biomass yield and thereby contribute to the apparent high GAR value.

Additionally, under aerobic conditions some less energetically efficient enzymes are expressed at higher levels than their more energetically efficient counterparts. This includes two NADH dehydrogenases, Ndh (SO3517) and Nqr (SO1103–1108). These both show higher expression and higher relative changes in expression (aerobic versus O_2_-limited conditions) than the *nuo* genes, which encode for the more energetically efficient proton-translocating NADH dehydrogenase. Likewise, the ABC-sulfate transporter (SO3599–3602) shows higher expression than the proton symport sulfate transporters SulP (SO2286 or SO3553), the latter being more energetically efficient.

### Comparing Model Predictions to Qualitative Growth Phenotypes

Although *S. oneidensis* MR-1 has been considered to be rather limited in terms of the compounds it can use as carbon and energy sources [36], its genome analysis implies that this bacterium has pathways for utilization of different amino acids, nucleosides, fatty acids, and C_1_–C_3_ compounds [37]. To further investigate *S. oneidensis* MR-1's metabolic capabilities, we used flux balance analysis (FBA) to identify metabolites that could be used as sole carbon sources under aerobic conditions, and then experimentally tested some of those predictions. Thirty-three compounds were predicted to be able to support *S. oneidensis* MR-1 aerobic growth, indicating that all the necessary transporters and catabolic enzymes are present in the genome. About half of these were reported previously or were found in this study to be able to support growth of *S. oneidensis* MR-1 experimentally (see Table S5 for details).

Some of the predicted carbon sources are amino acids, and while there have been reports of *S. oneidensis* MR-1 growing on mixtures of amino acids [38], there have been no studies investigating which individual amino acids support growth as sole carbon and energy sources. We subsequently screened eight individual amino acids and found that glutamate, glutamine, threonine, and serine were able to support *S. oneidensis* MR-1 growth, while alanine, aspartate, asparagine, and glycine could not (Table S5). This indicates that for the latter four amino acids, regulatory or kinetic limitations likely prevent their use as sole carbon and energy sources, because these bacteria appear to have the enzymes and transporters needed to degrade them.

The inability of *S. oneidensis* MR-1 to grow on glycine was investigated further. The model predicted that MR-1 could metabolize glycine by first using the glycine cleavage system to convert glycine into 5,10-methylenetetrahydrofolate (mlthf), CO_2_ and NH_4_, and then combining mlthf with another glycine molecule to produce serine (by serine hydroxymethyltransferase, *glyA*) which can then be deaminated into pyruvate. Further experiments revealed that (i) addition of glycine restored aerobic growth on lactate of *S. oneidensis* MR-1 *glyA* deletion mutant (see Figure S2 and S3), which is unable to synthesize glycine from serine, and (ii) utilization of glycine as the sole source of nitrogen was mainly dependent on glycine cleavage system (see Text S1 and Figure S4) indicating that glycine can be taken up and metabolized. Based on these results, we concluded that *S. oneidensis* MR-1 is unable to use glycine as a sole carbon source, possibly due to transport limitations and/or insufficient activity of serine hydroxymethyltransferase (in the glycine to serine direction) due to kinetic or transcriptional regulatory limitations.

During our evaluation of *S. oneidensis* MR-1's capability to use carbon sources, we found four cases where the model did not correctly predict the use of carbon sources that had been shown to support growth experimentally, including threonine, adenosine, deoxyadenosine, and inosine. We found that adding a secretion reaction for hypoxanthine to the metabolic model would allow the model to predict growth on adenosine, deoxyadenosine, and inosine, while the addition of a threonine transport reaction enabled the model to accurately predict growth on threonine.

### Comparison between Predicted and Experimental Biomass Yields

**Figure 3:**
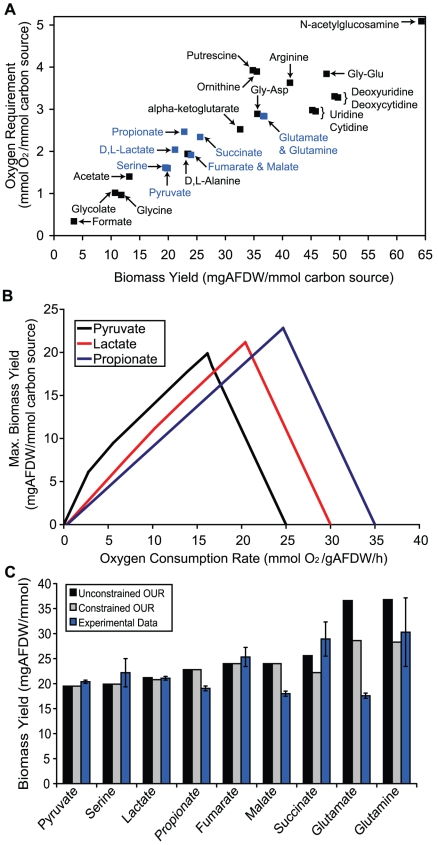
Oxygen requirements for maximal biomass production. Panel A shows the maximal biomass yield and oxygen requirements needed to achieve the maximum biomass yields for 30 of the 33 model predicted carbon sources (not shown are fatty acids which lie outside of the region shown, see Table S5 for complete list of values). Blue points correspond to carbon sources that were evaluated experimentally. Panel B shows how the maximum biomass yield is affected as the O_2_ consumption rate is increased and decreased from its optimal value. All three carbon sources have the same number of carbon atoms, but pyruvate requires the least amount of oxygen and under oxygen limitations will have higher biomass yields than the other two carbon sources. All simulations were done assuming a carbon source consumption rate of 10 mmol ATP/(g AFDW•h). Panel C compares calculated biomass yields with experimental biomass yields as estimated from batch growth in a microplate reader. The model predictions were made assuming a carbon source consumption rate of 10 mmol ATP/(g AFDW•h) with either an unconstrained OUR or a maximum OUR of 20 mmol ATP/(g AFDW•h), based on maximal estimates for *E. coli* [47].

We also used FBA to evaluate the quantitative accuracy of growth phenotype predictions. Biomass yields (g AFDW/mmol carbon source) were calculated by constraining the consumption rate for carbon sources and identifying flux distributions that maximize growth rate using FBA. As with all previous simulations, the ratio of fluxes between the cytochrome oxidases was constrained so that 2.8H^+^ are translocated for each pair of electrons that move from ubiquinol to O_2_. Figure 3A shows the calculated maximal biomass yields for thirty of the thirty-three model predicted carbon sources identified above and the corresponding O_2_ requirements (mmol O_2_/mmol carbon source) needed to achieve the maximal biomass yield (see Table S5 for complete details; the three fatty acids fall outside the region shown in Figure 3A). The O_2_ requirements were normalized to mmol carbon source to be consistent with the predicted biomass yields (mgAFDW/mmol carbon source) and to reflect the amount of O_2_ needed to convert a fixed amount of substrate into biomass. Substrates such as putrescine, ornithine, propionate, and acetate have the highest ratios of O_2_ requirement to biomass yields, while the nucleosides (cytidine, uridine, deoxyuridine and deoxycytidine) have the lowest ratios.

To further evaluate the sensitivity of the biomass yields to O_2_ consumption rates, we fixed the substrate consumption rate for three different C_3_ compounds and constrained the O_2_ consumption rate to different values. The corresponding calculated biomass yields were significantly affected by the O_2_ consumption rate (Figure 3B). If O_2_ consumption rates are too high, no biomass can be produced, as all carbon is oxidized to CO_2_, and no biomass can be produced without O_2_ (in agreement with the inability of MR-1 to grow fermentatively). In addition to the O_2_ consumption rate, the calculated biomass yields can also be sensitive to biomass composition measurements used to formulate the biomass reaction. To evaluate the effects of biomass composition on calculated biomass yields, the protein, DNA, RNA, and glycogen abundances were independently altered ±30% from their measured values, with corresponding reductions or elevations in the levels of other biomass components. The calculated biomass yields were most sensitive to changes in protein levels, but even a 30% decrease in protein abundance only led to a 2.5% increase in predicted biomass yield. Overall, the calculated biomass yields were more sensitive to changes in O_2_ consumption rates than to biomass composition.

Given this result, we compared our model predicted biomass yields, both with and without O_2_ consumption rate constraints, to experimental measurements. For most of the carbon sources, the experimentally measured biomass yields were within 15% of the model calculated values when restrictions were placed on O_2_ consumption rates (Figure 3C). The exceptions were growth on glutamate, succinate, propionate, and malate. Interestingly, succinate, propionate, and glutamate had three of the four highest predicted O_2_ requirements of the substrates experimentally tested (Figure 3A) indicating that the calculated biomass yields will be more sensitive to the maximum O_2_ consumption rate used in the simulation. The low experimental biomass yields may indicate there were oxygen limitations in the microplate experiments or that *S. oneidensis* MR-1 has not evolved to efficiently use these carbon sources. Performing additional experimental measurements of substrate and O_2_ consumption rates will likely improve model predictions of biomass yields for these three substrates.

### Flux Analysis of Lactate-Limited Aerobic Growth

FBA was used to predict which metabolic pathways are likely operational during lactate-limited aerobic growth in a chemostat. Simulations were done for lactate-limited aerobic growth corresponding to a growth rate (μ) of 0.085 h^−1^ and assuming 2.8H^+^ translocated per electron pair transferred from ubiquinol to O_2_. Using the previously determined values for growth- and non-growth rate dependent ATP requirements (Figure 1A), the minimum lactate consumption rate consistent with μ = 0.085 h^−1^ was 4.08 mmol/g AFDW/h, which was in good agreement with our experimental measurement of 4.06 mmol/g AFDW/h. Multiple flux distributions could be identified with the same growth and lactate consumption rates, indicating that the FBA solution is not unique. Therefore, FVA was used to determine for each reaction the range of flux values that can still result in the maximum biomass yield.

As described earlier, optimal reactions are those that can be used to achieve the maximum biomass yield. Optimal reactions can further be classified as either optional or required depending on whether the reaction has to be used to achieve the maximum biomass yield. In some cases a zero flux through an optimal reaction can still result in an optimal solution (classified as optional reaction), while other optimal reactions are required to carry flux to achieve optimal biomass yield (classified as required reaction). For example, *S. oneidensis* MR-1 has two isocitrate dehydrogenases that use either NAD^+^ or NADP^+^ as electron acceptors. The model predicts that the NADP^+^-dependent enzyme is required for optimal biomass yield, while flux through the NAD^+^-dependent isocitrate dehydrogenase is optional. The periplasmic fumarate reductase is another example of an optional reaction, however, it is known to be inactive aerobically [25].

**Figure 4:**
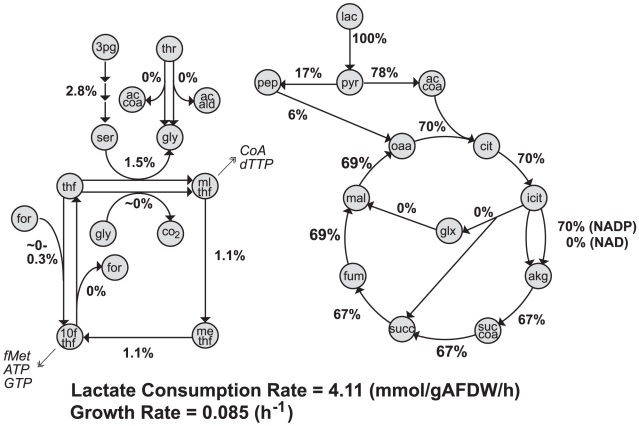
Model predicted flux values in central and C1 metabolism. The figure shows the range of flux values calculated using FVA that correspond to maximal biomass yields in lactate-limited aerobic growth when malate synthase, pyruvate formate lyase, NAD+ dependent isocitrate dehydrogenase, and fumarate reductase are constrained to be zero (see text for details). Flux values are reported as the percentage of the lactate consumption rate, 4.11 mmol/(g AFDW•h). Cellular growth rate was constrained to 0.085 h^−1^. Metabolite abbreviations are described in text and/or can be found in Table S3.

We have subsequently measured the specific activities for a variety of enzymes to compare against our model predictions (Table 1; see Text S2 for methods description). NAD^+^-dependent isocitrate dehydrogenase, malate synthase, and pyruvate formate lyase activities were not detected; therefore, solutions that carry flux through these reactions and the fumarate reductase reaction were subsequently excluded from the model. The additional constraints on these four fluxes caused a small increase (∼0.7%) in the lactate consumption rate that was needed to maintain the growth rate at 0.085 h^−1^ (and thus a small decrease in biomass yield); however, the re-calculated flux values were better resolved and had less individual variability (Figure 4). This illustrates how iterations of computation and experimentation can be used to better identify the pathways and enzymes that are important in particular growth conditions.

**Table 1:** Enzyme activity for cells grown in lactate-limited aerobic chemostat.

Enzyme Name	Specific Activity (Units/min/mg protein)
Pyruvate Dehydrogenase	5.85
Pyruvate Formate Lyase	ND
Isocitrate Dehydrogenase (NAD dependent)	ND
Isocitrate Dehydrogenase (NADP dependent)	0.42
Malate Dehydrogenase (NAD dependent)	0.33
Malate Synthase	ND
Isocitrate Lyase	0.085

ND: not detected.

The model also predicts that during lactate-limited aerobic growth tetrahydrofolate (thf) molecules carrying one-carbon units (5,10-methylenetetrahydrofolate, mlthf; 5,10-methenyltetrahydrofolate, methf; and 10-formyltetrahydrofolate, 10fthf) are produced during conversion of serine to glycine (by serine hydroxymethyltransferase - *glyA*, SO3471) and possibly from formate, but not from the degradation of glycine into CO_2_ and NH_4_^+^ (by the glycine cleavage system—*gcvTHP* and *lpdA*; SO0779–0781 and SO0426) (Figure 4). These one-carbon units are used to synthesize a number of biomass components, including CoA, dTTP, ATP, GTP, and formyl-methionine (fMet). Even though the *S. oneidensis* MR-1 genome encodes for two potential ways to generate mlthf under aerobic conditions, the model calculations suggest that the glycine cleavage system is not essential. Experiments confirmed this prediction, as a mutant defective in the glycine cleavage system had a similar growth phenotype on lactate as did the wild-type (Text S1).

As noted earlier, the model predicts that for maximal biomass yields one-carbon units are mainly made by serine hydroxymethyltransferase (*glyA*), and its corresponding reaction (ser+thf↔gly+mlthf) operates in the forward direction (Figure 4), indicating that glycine is made from serine, instead of threonine being degraded into glycine and then converted into serine (see Figure S5). Experimental assessment of a Δ*glyA* mutant found that *glyA* is essential for aerobic growth on lactate (Figure S2 and Figure S6) indicating that serine hydroxymethyltransferase is used in the production of either serine or glycine and one-carbon units. *S. oneidensis* MR-1 has two alternate metabolic routes to produce glycine from threonine in the absence of *glyA* (Figure 4 and Figure S5); as a result, the model only predicts a lethal phenotype for a Δ*glyA* mutant if these alternate routes are removed from the network. Given that the Δ*glyA* mutant was experimentally unable to grow on lactate under aerobic conditions, it is likely that these alternative enzymes for glycine production were not expressed or active. The model predicts that in order to restore growth of the Δ*glyA* mutant one of these threonine to glycine routes would need to be available or alternatively glycine would need to be added to the medium. In fact, experiments demonstrated that the addition of either threonine or glycine to M1 medium with lactate restored growth of Δ*glyA* strain, whereas serine addition did not (see Text S1 and Figure S3). These experimental observations are both in agreement with *in silico* assessments, assuming that threonine addition increases the expression of the enzymes that convert threonine to glycine. Taken together, these results confirm the model prediction that reversible serine hydroxymethyltransferase operates in the serine to glycine direction in *S. oneidensis* MR-1 cells *in vivo*. This agrees with recent findings based on ^13^C labeling experiments in carbon-limited aerobic chemostats [39].

## Discussion

In this work, we presented the development of a metabolic model for the facultative dissimilatory metal-reducing bacterium *S. oneidensis* MR-1, an organism with applications in bioremediation, energy-generating biocatalysis, and chemical production. The model served as a framework to provide context for experimental data, to quantitatively evaluate experimental observations, and to generate hypotheses about metabolic network utilization and physiological capabilities. Here, we were able to use a combination of modeling and experimentation to identify pathways that are used under lactate-limited aerobic conditions, and those that are not used. These unused pathways include threonine degradation (to produce glycine), the glyoxylate shunt, and, unexpectedly, the more energetically efficient components of the aerobic respiratory chain.

Based on our analysis of lactate-limited growth at different dilution rates, *S. oneidensis* MR-1 appeared to have an unusually high growth rate dependent ATP requirement (GAR). Our model directly accounts for the energy requirements needed to generate biomass components including macromolecule polymerization. The remaining GAR in our model may be attributed to membrane processes (proton leakage), protein and mRNA turnover, and other unknown costs [40]. The GAR for *S. oneidensis* MR-1 (220 mmol/gAFDW) is 2.5 times higher than that reported for *Bacillus subtilis* (88 mmol/gDW, when protein polymerization costs are removed, the highest reported value for GAR). There are a number of possible explanations for the observed high GAR, including flux through futile cycles and use of less energetically efficient enzymes, such as components in the aerobic electron transport chain. Accounting for these inefficiencies in the model would reduce the calculated GAR and NGAR values (see details below).

Futile cycles have been shown to be active in other bacteria [41], [42], and it is possible that they are active in *S. oneidensis* MR-1 under the experimental conditions we tested. Futile cycles have been suggested to be beneficial for increasing network robustness and sensitivity, generating heat, reducing build-up of toxic intermediates, and providing competitive advantages in energy rich environments [43], [44]. Here, we used optimization to not only calculate solutions corresponding to maximum biomass yields, but also to investigate suboptimal solutions. We developed a new optimization-based approach to identify cycles, such as futile cycles, that does not require the calculation of extreme pathways [45]. This makes them easier to enumerate because it does not require implementation of the model into another software format. Next, by analyzing fluxes through individual metabolic reactions, the reactions were classified into different groups based on how non-zero fluxes affect cellular growth rates. By comparing the associated genes with high-throughput data (such as gene expression or proteomic data), hypotheses can be made about which pathways may cause suboptimal growth phenotypes. As a result, we were able to identify potential futile cycles, involving pyruvate kinase and malic enzymes, that may be active based on analysis of existing gene expression data. Subsequent removal of either malic enzyme led to ∼25% improvement in biomass production. However, further calculations revealed that the level of futile cycling has to be three times the lactate consumption rate to reduce the GAR value to ∼80 (a value similar to that reported for *B. subtilis*). Therefore, it seems unlikely that futile cycling is the only explanation for the high apparent GAR value.

Like many other bacteria, *S. oneidensis* MR-1 has a branched electron transport chain. The *S. oneidensis* MR-1 genome contains annotated genes for (i) three cytochrome oxidases, which results in either translocation of 2 H^+^/2e^−^ (Cyd, SO3285–3286) or 6 H^+^/2e^−^ (Cco, SO2361–2364; or Cox, SO4606–4607, SO4609) as electrons move from ubiquinol to O_2_, (ii) two NADH dehydrogenases translocating either 0 H^+^/2e^−^ (Ndh, SO3517) or 4 H^+^/2e^−^ (Nuo, SO1009–SO1021), and (iii) three NADH dehydrogenases that translocate 2 Na^+^/2e^−^ (Nqr1, SO1103–1108; Nqr2, SO0902–0907; and Rnf, SO2508–2513). As a result, the transfer of a pair of electrons from NADH to O_2_ can result in the translocation of 2 to 10 H^+^ across the cytoplasmic membrane, depending on what enzymes are used. In all aerobic simulations, we constrained the flux ratios between the cytochrome oxidases such that they result in the translocation of 2.8 H^+^/2e^−^ based on experimental data [25]. However, no constraint was placed on the ratio between the NADH dehydrogenase fluxes because no data were available regarding their relative usage. To produce the maximum amount of biomass the model predicts that Nuo (encoding the proton-pumping NADH dehydrogenase) would be used and not Ndh, Nqr1, Nqr2, and Rnf. However, if Nuo is inactive so that there is no flux through its associated reactions, the estimated GAR drops from 220 to 119. Interestingly, *S. oneidensis* MR-1, *Shewanella benthica* KT99, and *S. woodyi* are the only strains among 20 sequenced *Shewanella* strains analyzed that have *nuo* orthologs and it would be expected that these three strains would grow more efficiently than those strains missing *nuo* orthologs. However, the presence of *nuo* orthologs in *S. oneidensis* MR-1 did not confer any growth advantage over two *Shewanella* strains that do not contain *nuo* orthologs, *S. putrefaciens* CN32 and *Shewanella* sp. strain W3-18-1. These latter two strains (CN32 and W3-18-1) both had higher growth rates and biomass production (as indicated by optical density measurements) than *S. oneidensis* MR-1 when grown on lactate in aerobic cultures [2]. These results support the suggestion that the Nuo proton-pumping NADH dehydrogenase may not account for a significant fraction of NADH oxidation in *S. oneidensis* MR-1 cells under the growth conditions tested. Additionally, data published by other researchers showed that aerobic growth of *E. coli* mutants with disabled Nuo-type NADH dehydrogenase on minimal medium supplemented with mannitol or glycerol was undistinguishable from wild-type cultures [46], implying that use of this Nuo NADH dehydrogenase type may be condition-dependent for bacteria other than *Shewanella*.

Further computational analysis revealed that if all three of the most efficient H^+^ pumping enzymes are not active (Nuo, Cco, and Cox), then at most, 2H^+^/2e^−^ can be translocated via the electron transport chain and the GAR drops to 81. In this calculation the constraint fixing 2.8H+ per electron pair transferred from ubiquinol to O_2_ was not included in the simulation. Similarly, the NGAR in this case also dropped from 1.03 to 0.47, the latter value being closer to NGAR value reported for *Geobacter* [30]. By not utilizing Cco and Cox, only 2H^+^/2e− can be translocated across the membrane via cytochrome oxidase activity, which disagrees with previous experimental measurements [25]. However, these experimental results depend on the growth conditions of the cells prior to measurements being made [25], and these growth conditions may not be consistent with those used in our experiments. Therefore, we hypothesize that MR-1 does not use the most energetically efficient components of its electron transport chain under the conditions tested in this study, and that this is likely the main reason for the high estimated GAR value. This was supported by the subsequent phenotyping of a single Cox (*ΔSO4606*) deletion mutant and a double Cox and Cco (*ΔSO4606/ΔSO2361*) deletion mutant, both of which exhibited growth rates in batch culture that did not differ significantly from the wild-type strain, and in fact grew to higher optical densities than wild-type cultures (Figure S1B). We should also note that while changes in the proton translocation efficiency of the electron transport chain will affect the calculated GAR and NGAR values, this should not significantly affect other calculations such as biomass yields, flux distributions and reaction classification (e.g. optimal and suboptimal reactions.) This is because the effect of the lower proton translocation efficiency will be canceled out by a lower GAR and NGAR value. When fewer protons are translocated across the membrane, less ATP is produced by ATP synthase, but less ATP is subsequently needed for GAR and NGAR, keeping the net ATP production the same.

Our findings that (i) the energetically efficient cytochrome oxidases are not utilized and that (2) futile cycles involving malic enzymes likely operate during aerobic growth on lactate indicates that *S. oneidensis* MR-1 does not achieve maximal biomass production under the highly aerobic conditions tested in this study. Given that the organism is found in anoxic and suboxic environments it is possible that it is not accustomed to the carbon and oxygen rich environments we tested here, and that adaptive evolution of this organism under the conditions used here may lead to improved biomass yields and metabolic efficiency, as has been observed for *E. coli* [47]. The classification of optimal and suboptimal reactions done here is based primarily on energetic efficiency, and does not account for the kinetic properties of enzymes or the relative costs of enzyme production [48]. The utilization of less energetically efficient enzymes that are more kinetically efficient may provide a competitive advantage in terms of flux per unit enzyme when substrate concentrations are low. For example, in *E. coli* the cytochrome *bd* oxidase has a higher affinity for oxygen making it more beneficial to use under low oxygen concentrations even though it is less energetically efficient [22]. Trade-offs between growth yields and rates have been theorized and demonstrated [49], [50], where increased rates are accompanied by decreased yields. Thus, *S. oneidensis* MR-1 may have evolved in its natural environment to achieve high rates rather than yields.

The integration of experimental and modeling results is extremely valuable to advance model development and biological discovery. In cases where there is agreement between data and model predictions, the models can be used to help explain observed cellular behavior (e.g., what metabolic pathways are being used under a given condition), analyze experimental data, or engineer metabolism for specific applications. For example, the model predicted that the TCA cycle is important for aerobic growth on lactate by *S. oneidensis* MR-1, a finding that was also confirmed experimentally. Model predictions of TCA cycle fluxes in cells grown in a lactate-limited aerobic chemostat (with a growth rate of 0.085 h^−1^) showed that a significant fraction (∼70%) of lactate may be oxidized by TCA cycle (Figure 4). In agreement with the model calculations, cell-free extracts of *S. oneidensis* MR-1 grown in lactate-limited aerobic chemostat (D = 0.095 h^−1^) displayed a high specific activity of pyruvate dehydrogenase (Table 1). Additionally, disruption of the TCA by deletion of the E1 subunit of pyruvate dehydrogenase or E2 subunit of alpha-ketoglutarate dehydrogenase (KGDH) totally impaired the ability of MR-1 to grow aerobically on any tested single substrate in batch cultures (see Figure S7 and Text S1). Interesting, the KGDH mutant was unable to grow aerobically even in rich medium, which is in contrast to *E. coli* KGDH mutants which retain their ability to grow aerobically in rich (LB) [51] and glycerol minimal media [52].

As more cycles of model prediction and experimental testing are carried out, both the model and our knowledge of *S. oneidensis* MR-1 metabolism will improve. Integrated models of metabolism and regulation for this organism (as has been done with *E. coli* [53] and *Saccharomyces cerevisiae* [54]) will undoubtedly lead to improved model predictions. Such predictions can be used to design strains with desired phenotypes, to provide a better understanding of which enzymes or pathways are important for survival and growth in a particular environment, and can be used for understanding of organism ecology. The developed model can also be used as a template for developing models of other *Shewanella*, particularly those that have been sequenced, as well as other organisms that have orthologs to genes included in the *S. oneidensis* MR-1 model. For example, many of the genes that were computationally determined to be essential for growth in *S. oneidensis* MR-1 are highly conserved in other *Shewanella* species, indicating a conserved set of core metabolic processes and capabilities across these bacteria.

## Materials and Methods

### Bacterial Strains and Growth Media

The strains of *S. oneidensis* MR-1 used in this study are listed in Table S6. Wild-type and mutant strains were routinely cultured at 30°C in tryptic soy broth (TSB; pH 7.4) [55] or in modified M1 medium (pH 7.0) of the following composition: piperazine-N,N′-bis(2-ethanesulfonic acid (30 mM and 3 mM for batch and chemostat cultivation, respectively), 28 mM NH_4_Cl, 4.35 mM NaH_2_PO_4_•H_2_O, 30 mM NaCl, 3 mM MgCl_2_•6H_2_O, 1.34 mM KCl, 6.8 µM CaCl_2_, 1 µM Na_2_SeO_4_, and 10 ml each of 10× Wolfe's vitamin solution and 10× mineral solution [56]. The modified M1 medium used for controlled chemostat cultivation was additionally supplemented with 18mM lactate and 10 µM ferric nitrilotriacetic acid [Fe(III)-NTA]. *S. oneidensis* MR-1 growth was tested using 18 organic compounds, containing from 1 to 5 carbon atoms (alanine, asparagine, aspartate, glutamate, glutamine, glycine, serine, threonine, formate, acetate, ethanol, pyruvate, lactate, propionate, succinate, fumarate, malate, and α-ketoglutarate) at concentration 40 mM unless stated otherwise. To grow *S. oneidensis* MR-1 under anaerobic conditions, NaCl was excluded from M1 medium, and sodium fumarate was added as an electron acceptor to final concentration 35 mM. Anaerobiosis was achieved by extensively purging medium with pure N_2_.

### Genetic Manipulations

In-frame deletion mutagenesis in *S. oneidensis* MR-1 was performed using a previously described method [57], [58]. The sucrose selection step for obtaining SO1931 and SO4606/SO2361 deletion mutants was performed under anaerobic conditions at 25°C using plates containing modified M1 medium supplemented with 20 mM D, L-lactate, 30 mM sodium fumarate, 10% LB, and 1.5% agar. Table S7 contains list of primers used in this study.

### Chemostat and Batch Cultivation

A 15-liter New Brunswick Bioflow 3000 reactor (New Brunswick Scientific, Edison, NJ) operated at a 7-liter working volume (modified M1 medium supplemented with 18 mM D,L-lactate) at 30°C was used to grow chemostat cultures of *S. oneidensis* MR-1 in modified M1 media supplemented with 18mM lactate. The gas flow rate and agitation were kept at 3.5 liters/min and 350 rpm, respectively, and the dissolved oxygen tension (DOT) was maintained at 20% or 1% of air saturation by automatically changing the ratio of N_2_ and air in the gas mix. The pH was maintained at 7.0 by addition of 2 M HCl, and composition of incoming and off-gas was constantly monitored by an in-line mass spectrometry based gas analyzer MGA iSCAN (Hamilton Sundstrand, Pomona CA).

Culture growth was constantly monitored by measuring and recording optical density using a custom-made system. For this purpose, a fluorescent lamp was placed near the bioreactor wall, and a photodiode (Silicon Solar Cell, Model 276-124, RadioShack) was secured on the opposite side of the wall to quantify transmitted light. Photodiode voltages were recorded by a Keithley Model 2700 multimeter (Keithley Instruments, Inc., Cleveland, OH) using the ExceLinx data logging software (Microsoft Corp.). Additionally, 5- to 10-ml samples were periodically taken from the reactors for OD_600_ measurements using Spectronic® model 20 GENESIS™ VIS spectrophotometer (Spectronic Instruments, Rochester NY, USA), and/or further analyses when necessary.

Reactors were inoculated with 1mL/L of overnight culture grown in TSB and maintained in batch mode until late-logarithmic stage. Continuous cultures were started by pumping medium of the same composition at desired dilution rate (D). Samples for further analysis were taken after at least five volume changes at steady-state conditions. Steady-state achievement was inferred from stability of the following parameters: OD_600_ (no more than 3% variation between measurements), acid addition rate, O_2_ concentration in incoming gas, and O_2_ and CO_2_ concentrations in the off-gas. Samples for the following measurements were taken from reactors: (i) ash-free dry organic weight (AFDW), (ii) biomass composition, (iii) organic acids composition. Sampling procedure took no more than 4 minutes.

Batch experiments were performed at 30°C in an Infinite M200 (Tecan, Männedorf, Switzerland) or Bioscreen-C (Growth Curves USA, Piscataway, NJ) microplate reader, or crimp-sealed serum bottles placed on a rotary shaker (Innova 4900, New Brunswick Scientific, Edison, NJ) at 30°C and 150 rpm. Growth was monitored by measuring the cultures' optical density at 600 nm (OD_600_). Cells were grown with three or more replicates in 96- or 100-well plates at 30°C. M1 minimal media supplemented with 40 mM carbon source was used in growth phenotyping experiments. Absorbance readings were taken every 15 minutes at 600 nm. A standard curve was used to convert absorbance readings in a microplate reader to a standard spectrophotometer with a 1-cm pathlength. Alternately, cultures were grown in shaken (150 rpm) 70 mL crimp-sealed serum bottles containing 10 mL of medium. If necessary, the OD measurements were converted to biomass concentration (gAFDW/L) by multiplying by 0.69 using previously established correlations between OD_600_ and cell concentration (g AFDW/L). All batch experiments were repeated at least 3 times, and the standard deviation of triplicate cultures never exceeded 6%, unless indicated otherwise by error bars. Under conditions used to grow cells in M1 medium supplemented with 18–20 mM lactate all substrate was consumed when cultures entered stationary phase.

### Analytical Methods

To measure biomass ash-free dry weight (AFDW), a known volume of cultural liquid was centrifuged (11,000×g, 4°C), supernatant discarded, pellet thoroughly resuspended in deionized water, transferred into a pre-weighed aluminum dish and, using Sartorius MA100 Mositure Analyzer (Sartorius Goettingen, Germany), dried at 105°C to a constant weight (DW1). The dried biomass was then combusted at 600°C for 12 h and weighed again (DW2). The biomass concentration (gAFDW/L) was calculated as follows: (DW1–DW2)/volume.

For the purpose of this work, biomass was considered to include the cell pellet following centrifugation and extracellular polymers present in the supernatant. Bacteria can produce substantial amounts of extracellular polymers (protein and carbohydrates) as well as membrane vesicles containing the above as well as membrane lipids. For the initial stages of metabolic modeling, the location of cell constituents is not critical but it is important to capture all biomass components in order for the model to be accurate. Therefore, biomass composition included analyses of culture liquid (CL) without preliminary fractionation. In all cases, a method of standard additions was used to quantify polymers in known CL volume [59]. Total protein, reducing carbohydrates, RNA, and DNA were assayed using previously described methods [60], [61]. The total mass of lipids was extracted from a known volume of freeze-dried CL and analyzed by Global Lipidomics, LLC (Text S2). Amino acid composition of biomass protein was analyzed using reversed-phase high-performance liquid chromatography (Text S2). Organic acids in cultural liquid were filtered through 0.22-µm-pore-size Millex-GP membrane filter (Millipore) and quantified by HPLC as described previously [2].

### Metabolic Network Reconstruction iSO783

The *S. oneidensis* MR-1 metabolic network was reconstructed in SimPheny (Genomatica, San Diego, CA) initially using an automated model building process [62] by comparing its genome sequence to those of previously modeled organisms. Each gene in *Shewanella* was the subject of a reciprocal BLAST search against genes from other microorganisms with well-curated metabolic reconstructions. Metabolic reactions and gene to protein to reaction (GPR) associations from other models were included in the draft *Shewanella* reconstruction if a good reciprocal best hit could be found in *S. oneidensis* MR-1.

This draft reconstruction was manually reviewed to exclude and include additional genes and reactions. GPR associations in the draft reconstruction were reviewed to confirm that the gene's annotated functions were consistent with the associated reactions. Genes not included in the draft reconstruction were also evaluated and included in the reconstruction if their products were predicted to carry out metabolic functions. A large fraction of the anaerobic electron transport chain reactions were added to the draft reconstruction at this stage because these reactions do not occur in other reconstructed organisms. Additional reactions were added as necessary to produce known biomass constituents or utilize known growth substrates.

The final reconstruction includes organism-specific reactions associated with macromolecular synthesis, which were incorrect in the draft reconstruction because they differ from other reconstructed organisms. The structure of lipopolysaccharide had been previously determined for MR-1 [63], and this was used to generate reactions for the biosynthesis of this biomass component. The measured fatty acid content on phospholipids (measured in this study and in a previous publication [3]) was also used to generate MR-1 specific phospholipid biosynthesis reactions. Measured amino acid levels were used to create a protein biosynthesis reaction. The monomeric compositions of RNA and DNA were estimated based on the GC content for MR-1 and used in the RNA and DNA biosynthesis reactions.

A biomass reaction was generated from measured biomass compositions (protein, RNA, DNA, phospholipids, and carbohydrate) for MR-1. Cells grown in a lactate limited chemostat (D = 0.095 h^−1^) were used for biomass composition analysis. The measured biomass composition was then used to generate a biomass reaction, which is included in the metabolic model. This biomass reaction specifies the amount of biomass components (mmol) needed to synthesize 1 g AFDW of cells. Lipopolysaccharide and peptidoglycan abundances (g/g AFDW) were estimated based on values reported for *E. coli* [19]. In addition, soluble pools for acetyl-CoA, succinyl-CoA, putrescine, spermidine, UDP-glucose, 5-methyltetrahydrafolate, CoA, FAD, NAD(P), NAD(P)H, and AMP were included based on measurements for *E. coli* [40].

To estimate growth- and non-growth rate dependent ATP requirements (mmol ATP/(g AFDW•h)), a series of *S. oneidensis* MR-1 chemostat cultures were grown at different dilution rates and the lactate consumption rates (mmol/(g AFDW•h)) were measured. The growth and non-growth rate dependent ATP requirements were calculated by the metabolic model using this data as described previously [26]. Briefly, for each dilution rate the cell growth and lactate consumption rates were constrained to their measured values and maximum rate of ATP hydrolysis is calculated using the metabolic model. A linear curve fit between the maximum ATP hydrolysis rate and dilution rate was then used to calculate the energy requirements, where the slope is the growth rate dependent ATP requirement and the intercept is the non-growth rate dependent ATP requirement (or ATP maintenance requirement).

### Constraint-Based Analysis

A constraint-based model was developed based on the metabolic reconstruction for MR-1. These models include three types of constraints, the first is a steady-state mass balance constraint, where the production rate minus the consumption rate for each metabolite must equal zero. These steady-state mass balance constraints can be represented as:
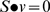
(1)where 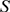 is a matrix of stoichiometric coefficients for the reaction network (rows correspond to metabolites and columns to reactions) and 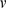 is a vector of fluxes corresponding to the variables in the model. The other two types of constraints place restrictions on the values individual fluxes (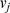) can take:
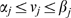
(2)Thermodynamic constraints can be imposed on irreversible reactions by setting the corresponding values for 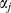 to zero. Additionally, enzyme capacity constraints can be imposed by specifying 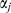 and 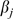 appropriately.

In most cases the linear system of equations is underdetermined so there are multiple flux distributions that satisfy the imposed the constraints. FBA uses optimization to identify flux distributions that maximize or minimize a given objective function [15]. For example, FBA can be used to find a flux distribution with maximizes flux through the biomass reaction. FVA determines the range of values each flux can take that are consistent with a given set of constraints [33]. Here, each flux is maximized and minimized individually. FVA can be used to find the range of flux values that are possible across alternative optimal solutions (by adding a constraint that the FBA objective function has to be equal to the maximum value determined by FBA) or across suboptimal solutions (by not including any constraint on the value for the FBA objective function). All optimization simulations were done using GAMS (GAMS Development Corporations, Washington DC).

### Enumeration of Cycles

We developed an optimization-based approach to calculate cycles (such as futile cycles, transhydrogenase cycles, and circulations) in constraint-based models of metabolism. All exchange fluxes are constrained to be zero so that no metabolites can enter or exit the system. To find futile cycles an artificial ATP synthesis (ATPS) reaction is added (ADP+P_i_+H^+^→ATP+H_2_O) and its flux is constrained to be positive; this ensures that a futile cycle must take on non-zero fluxes to hydrolyze the ATP that is produced by ATPS. FVA using these constraints can be used to identify all reactions that can participate in futile cycles. To find individual futile cycles, a mixed-integer linear programming problem was formulated (Eq. 3–8) to minimize the number of reactions included in the cycle (Eq. 3), where 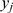 is a decision variable that indicates whether a flux is zero (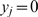) or non-zero (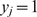) and *n* is the total number of reactions in the network. If the flux through the reaction is non-zero the corresponding value of the decision variable, *y_j_*, must be one (Eq. 5). The fluxes and decision variables must satisfy the mass balance (Eq. 4), thermodynamic, enzyme capacity constraints (Eq. 5), and positive ATPS flux constraint (Eq. 6, where 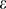 is a positive number— we used a value of 20 in the futile cycle calculations). To find additional cycles, integer-cut constraints can be added (Eq. 7) which ensure that the same solution is not revisited and that a new solution is not a combination of previous solutions [64], where 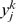 indicates whether reaction *j* was used in the previous iteration *k*. The problem can be solved repeatedly until it becomes infeasible, indicating that there are no more cycles.
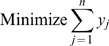
(3)

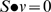
(4)

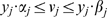
(5)

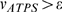
(6)

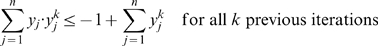
(7)

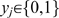
(8)Other types of cycles can be found using the same approach where Eq. 6 is changed to force selection of other cycles of interest (see Text S3 for more details). For example, transhydrogenase cycles can be found where the net effect of the cycle is NADH+NADP→NAD+NADPH, by adding an artificial reaction that undoes this reaction (NAD+NADPH→NADH+NADP) and constraining the corresponding flux to be positive. Such transhydrogenase cycles can be thermodynamically infeasible (if the intracellular ratios of [NAD]/[NADH]>[NADP]/[NADPH]) and would need to be eliminated from the network reconstruction. Cycles which result in no net change in metabolite levels (i.e. circulations [26]) can also be identified by replacing Eq. 6 with a constraint that the sum of the absolute flux values must be positive. More details for calculating all these types of cycles are presented in Text S3. Only twelve circulations and one transhydrogenase cycle (in the thermodynamically feasible direction: NAD+NADPH→NADH+NADP) were present in the *S. oneidensis* MR-1 network reconstruction.

## Supporting Information

Table S1 Biomass composition used to generate the biomass equation.
(0.02 MB XLS)

Table S2 Reactions and GPR associations in iSO783.
(0.28 MB XLS)

Table S3 Metabolites and their abbreviations used in iSO783.
(0.15 MB XLS)

Table S4 Reactions used in the smallest 130 futile cycles.
(0.02 MB XLS)

Table S5 The 33 model predicted carbon sources.
(0.02 MB XLS)

Table S6 Bacterial strains and plasmids used for this study.
(0.04 MB DOC)

Table S7 Primers used in this study.
(0.06 MB DOC)

Text S1 Summary of additional experimental results.
(0.06 MB DOC)

Text S2 Supplemental experimental methods.
(0.05 MB DOC)

Text S3 Methods for cycle calculations.
(0.13 MB DOC)

Figure S1 Growth dynamics of *S. oneidensis* MR-1 wild-type (filled circles), and selected deletion mutants. Panel A: *ΔSO3855* (open triangle) and *ΔSO4118* (open diamond). Panel B: *ΔSO4606* (open circle) and (filled triangle) double mutant *ΔSO4606/SO2363*. Cells were cultivated in 100-well plates in Bioscreen C; each well (550 µl total volume) received 100 µl of M1 medium supplemented with 20 mM D,L-lactate.
(0.10 MB PDF)

Figure S2 Growth of *S. oneidensis* MR-1 *ΔSO3471* mutants on lactate (45 mM) in M1 medium and organic acids concentration dynamics. Cells were added in the beginning to make final optical density 0.08 at 600 nm. Crimp-sealed serum bottles were used for cultivation.
(0.03 MB PDF)

Figure S3 Influence of glycine (A) and threonine (B) additions on maximal accumulation of *S. oneidensis* MR-1 *ΔSO3471* biomass on lactate (45 mM) in M1 medium. Biomass accumulation was expressed as optical density at 600 mn. Crimpsealed serum bottles were used for cultivation.
(0.12 MB PDF)

Figure S4 Growth dynamics of *S. oneidensis* MR-1 wild-type and selected deletion mutants. Wild-type (filled circle) and *ΔSO0781* (open circle) deletion mutant were grown on lactate (18 mM) or acetate (45 mM) in M1 medium supplemented with NH_4_Cl or glycine (10 mM) as the sole source of nitrogen. Crimp-sealed serum bottles were used for cultivation, starting OD_600_ values were 0.01 (A and B) and 0.003 (C).
(0.08 MB PDF)

Figure S5 Metabolic network for interconversion of C1-compounds and biosynthesis/degradation of relevant amino acids in *S. oneidensis* MR-1. The network was constructed based on genome annotation.
(0.10 MB PDF)

Figure S6 Growth of *S. oneidensis* MR-1 wild-type (A) and *ΔSO3471* mutant (B) in M1 medium supplemented with different compounds as sole sources of carbon and energy. Crimp-sealed serum bottles were used for cultivation, and starting OD_600_ value was 0.015 for all experiments.
(0.13 MB PDF)

Figure S7 Aerobic growth of *S. oneidensis ΔSO0424* cells in M1 medium supplemented with 18 mM D,L-lactate. 70 ml serum bottles containing 15 ml of medium were used. Periodically 0.7–0.8 ml samples were withdrawn to measure OD_600_ and organic acids in 0.22 µm filtrates of culture.
(0.07 MB PDF)
