# Supplementary material for: Constraint-Based Model of Shewanella oneidensis MR-1 Metabolism: A Tool for Data Analysis and Hypothesis Generation
Source: PLoS Comput Biol. 2010 Jun 24;6(6):e1000822. doi: 10.1371/journal.pcbi.1000822 (PMC2891590; doi:10.1371/journal.pcbi.1000822)
Supplement: Table S6 — Bacterial strains and plasmids used for this study. (0.04 MB DOC) [file pcbi.1000822.s006.doc]

**Table S6. Bacterial strains and plasmids used for this study.**

Strains and plasmids Description Reference

*Shewanella oneidensis*

MR-1 Manganese-reducing strain (Lake Oneida, NY) (Myers and Nealson, 1988)

SO0424 Δ*aceE* deletion derivative of MR-1 This work

SO1483 Δ*aceB* deletion derivative of MR-1 This work

SO1484 Δ*aceA* deletion derivative of MR-1 This work

SO2912 Δ*pflB* deletion derivative of MR-1 This work

SO0781 Δ*gcvP* deletion derivative of MR-1 This work

SO1931 Δ*sucB* deletion derivative of MR-1 This work

SO2363 Δ*ccoO* deletion derivative of MR-1 This work

SO3471 Δ*glyA* deletion derivative of MR-1 This work

SO3855 Δ*sfcA* deletion derivative of MR-1 This work

SO4118 Δ*SO4118* deletion derivative of MR-1 This work

SO4606 Δ*coxB* deletion derivative of MR-1 This work

*Escherichia coli*

EC100D pir-116 Host used for construction of pDS3.0 clones. Epicentre

F- *mcrA* *∆* (*mrr*-*hsdRMS*^-^ *mcrBC*) *φ*80d*lacZ∆*M15 *∆lacX*74 *recA*1 *endA*1 *araD*139 *∆* (*ara*, *leu*)7697 *galU galK* l- *rpsL* *nupG* pir-116

WM3064 λpir Host used for mating with MR-1. (Saltikov and Newman, 2003)

*thrB1*004 *pro thi rpsL hsdS lacZ∆*M15 RP4–1360 *∆*(*araBAD)567 ∆dapA*1341::[*erm pir*(wt)]

Top10 Host used for construction of pBBR1MCS-5 clones. Invitrogen

F- *mcrA ∆(mrr-hsdRMS-mcrBC) φ80lacZ∆M15 ∆lacX74 recA1 araD139 ∆(ara*-*leu)7697 galU galK rpsL (StrR) endA1 nupG*

Plasmids

pDS3.0 Suicide vector, Amp^r^, Gm^r^, *sacB*^+^ (Wan et al., 2004)

pDS3.0-*∆aceE* pDS3.0 containing sequences that flank *aceE* This work

pDS3.0-*∆aceB* pDS3.0 containing sequences that flank *aceB* This work

pDS3.0-*∆aceA* pDS3.0 containing sequences that flank *aceA* This work

pDS3.0-*∆pflB* pDS3.0 containing sequences that flank *pflB* This work

pDS3.0-*∆gcvP* pDS3.0 containing sequences that flank *gcvP* This work

pDS3.0-*∆sucB* pDS3.0 containing sequences that flank *sucB* This work

pDS3.0-*∆ccoO* pDS3.0 containing sequences that flank ccoO This work

pDS3.0-*∆glyA* pDS3.0 containing sequences that flank *glyA* This work

pDS3.0-*∆sfcA* pDS3.0 containing sequences that flank *sfcA* This work

pDS3.0-*∆SO4118* pDS3.0 containing sequences that flank *SO4118* This work

pDS3.0-*∆coxB* pDS3.0 containing sequences that flank *coxB* This work

pBBR1MCS-5 Used for complementation of deletions in MR-1 (Kovach et al., 1994)

*lacZ alpha*, Gm^r^

pBBR1MCS5-*aceE* Used for complementation of *aceE* -. This work

pBBR1MCS-5 containing native *aceE*

downstream to *lac* promoter.

pBBR1MCS5- *aceB* Used for complementation of *aceB* -. This work

pBBR1MCS-5 containing native *aceB*

downstream to *lac* promoter.

pBBR1MCS5-*aceA* Used for complementation of *aceA-*. This work

pBBR1MCS-5 containing native *aceA*

downstream to *lac* promoter.

pBBR1MCS5-*pflB* Used for complementation of *pflB* -. This work

pBBR1MCS-5 containing native *pflB*

downstream to *lac* promoter.

pBBR1MCS5-*gcvP* Used for complementation of *gcvP* -. This work

pBBR1MCS-5 containing native *gcvP*

downstream to *lac* promoter.

pBBR1MCS5-*sucB* Used for complementation of *sucB* -. This work

pBBR1MCS-5 containing native *sucB*

downstream to *lac* promoter.

pBBR1MCS5-*glyA* Used for complementation of *glyA* -. This work

pBBR1MCS-5 containing native *glyA*

downstream to *lac* promoter.

*E.coli* WM3064:

Reference:

Genetic identification of a respiratory arsenate reductase. Saltikov CW, Newman DK.   *Proc Natl Acad Sci U S A.* 2003 Sep 16; 100(19): **10983-10988**

pBBR1MCS-5:

Reference:

Kovach,M.E., Phillips,R.W., Elzer,P.H., Roop,R.M. and Peterson,K.M. pBBR1MCS: a broad host-range cloning vector. BioTechniques 16 (5), 800-802 (1994).

pDS3.0:

Reference:

Wan, X.F., Verberkmoes, N.C., McCue, L.A., Stanek, D., Connelly, H., Hauser, L.J. et al. (2004) Transcriptomic and proteomic characterization of the Fur modulon in the metal-reducing bacterium *Shewanella oneidensis*. *J Bacteriol* **186**: 8385- 8400.

*Shewanella oneidensis* MR-1*:*

Reference:

Myers, C.R., and Nealson, K.H. (1988) Bacterial manganese reduction and growth with manganese oxide as the sole electron acceptor. *Science* **240**: 1319–1321.
