# Supplementary material for: Constraint-Based Model of Shewanella oneidensis MR-1 Metabolism: A Tool for Data Analysis and Hypothesis Generation
Source: PLoS Comput Biol. 2010 Jun 24;6(6):e1000822. doi: 10.1371/journal.pcbi.1000822 (PMC2891590; doi:10.1371/journal.pcbi.1000822)
Supplement: Table S7 — Primers used in this study. (0.06 MB DOC) [file pcbi.1000822.s007.doc]

**Table S7. Primers used in this study.**

Primer name Nucleotide sequence*^a^*

∆SO0424 (*aceE-*)

0424_F-O 5’- CATTGCGTGAAGCCATTCA -3’

0424_5-O 5’- GAACAACTCTGGCAGAGTC -3’

0424_5-I 5’- AGCTACTTAGTTACCTAGGGAGCATATCTTCAGACATGACA -3’

0424_3-I 5’- ACCCTAGGTAACTAAGTAGCTCAGACAAGATCAATCCACAG -3’

0424_3-O 5’- GCTGGCATCACCAATATCAG -3’

0424_R-O 5’- GGTCAGCACTGATCATATC -3’

∆SO1483 (*aceB*^-^)

1483_F-O 5’- CGATTTATCCTGTGGCGAGC -3’

1483_5-O 5’- GGTTAACCATTTGGACTAAGC -3’

1483_5-I 5’- ACCCTAAGTAGGTAACTAGCTTGTGTTCCGTCATCTTCTCG -3’

1483_3-I 5’- ACCCTAAGTAGGTAACTAGCTGTTACGAGATGCTAACTGCTTA -3’

1483_3-O 5’- GATTGACACCGTTGCTCCAC -3’

1483_R-O 5’- ACTCGAACGCATTCAGTAC -3’

∆SO1484 (*aceA*^-^)

1484_F-O 5’- CGGTGTTTGAAATGGATGAA -3’

1484_5-O 5’- AGCGGTGACTATGGATACGC-3’

1484_5-I 5’- GACAGCACGTAATTGACCGCTCATAGTGTCACTCCTTTCTGTGTG -3’

1484_3-I 5’- GCGGTCAATTACGTGCTGTCGAGCAGTTTTAAGCCAACCA -3’

1484_3-O 5’- CATGACTCGTAGTCTTCATCTGG -3’

1484_R-O 5’- TACCATGGGGGACTGGTAAC -3’

∆SO2912 (*pflB^-^*)

2912_F-O 5’- ATCGTCGTTCCGTGCCTG -3’

2912_5-O 5’- GACTACAGTGAAGTGTTAATTGC -3’

2912_5-I 5’- ACTAGCTACCTAGTTACGGGATCGGTCATCGTACTAATACC -3’

2912_3-I 5’- ACCCGTAACTAGGTAGCTAGTCATTCACAAAAGGTCTGTAAG -3’

2912_3-O 5’- GACCACATAACGTATCCATG -3’

2912_R-O 5’- GCGAGCTGTAGAGCTGAG -3’

∆SO0781 (*gcvP*^-^)

0781_F-O 5’- CGTGTTCCTAGTTCAATTGG -3’

0781_5-O 5’- CTTTGTGCGCAACGGTAAAC -3’

0781_5-I 5’- ACTCACTCGCAGATGGGATGGGTCATGATAAACCTTG -3’

0781_3-I 5’- CATCCCATCTGCGAGTGAGTGAATAGTACGTTATTC -3’

0781_3-O 5’- CATATTCACCCTAGATATC -3’

0781_R-O 5’- GAATGCATTAAGTATTATG -3’

∆SO1931 (*sucB*^-^)

1931_F-O 5’- GGCATTCGAATATGGTTACGCAAC -3’

1931_5-O 5’- GTGATTGACCAGTTCTTATC -3’

1931_5-I 5’- TGGGCAGACACAACGCTACTCTCATGAAAAACGATCC -3’

1931_3-I 5’- TGTAGCGTTGTGTCTGCCCAGATCTGTAATCCAACTC -3’

1931_3-O 5’- GTGCAGGTTACCTTCAGTGGTG -3’

1931_R-O 5’- GATGGGTCGTGCATAGCTTTG -3’

∆SO2363 (*ccoO*^-^)

1931_F-O 5’- GCACTGATCGCACTGTACA -3’

1931_5-O 5’- GTATGGTGATGTCACTGATCCTA -3’

1931_5-I 5’- *GACCAGTGTGGCGTCATCAT*CGTGAAGATCGCTAACAAACCGA -3’

1931_3-I 5’- *ATGATGACGCCACACTGGTC*TCAGTCTTTGGGTCACGCACTCA -3’

1931_3-O 5’- CTTCGAGTGGTGTCGCCAAGTA -3’

1931_R-O 5’-TGCTACCACGTGCGTCAGA -3’

∆ SO3471 (*glyA^-^*)

3471_F-O 5’- GGATATTATCCAGATTGTGCG -3’

3471_5-O 5’- GCTTCAAGTTCGCCTTGCTC -3’

3471_5-I 5’- ACTACCTAGTTAGCTACGGGTGGATCATAATCTGCGATATTC -3’

3471_3-I 5’- ACCCGTAGCTAACTAGGTAGTCGCTCGTTTCCCTGTTTACG -3’

3471_3-O 5’- GCAGCTTAGCAATCGCCTCAC -3’

3471_R-O 5’- GAGTCATCCGCTTTTAGCGCA -3’

∆ SO3855 (*sfcA -*)

3471_F-O 5’- GCCGAGTATGGCGTAAACTG -3’

3471_5-O 5’- ACGCTGAAGTGATGCCAAAT -3’

3471_5-I 5’- AGATGCGTCCAATCCGTCTATATCGTCCATGGGTAACCTTT -3’

3471_3-I 5’- TAGACGGATTGGACGCATCTCGCACATCGTTCTAATTTCAG -3’

3471_3-O 5’- GGCGACGTTTATCCAACAAC -3’

3471_R-O 5’- AGGCTTAAAGCTGGGTGTCA -3’

∆ SO4118

4118_F-O 5’- GCGTTCGCAATGTTTC -3’

4118_5-O 5’- CCTTTCTGGTACGACTTC -3’

4118_5-I 5’- TGTCACGACCACAAAGGAGCCATTTTAACATCCTG -3’

4118_3-I 5’- GCTCCTTTGTGGTCGTGACAAGCAACTGTACGGTT -3’

4118_3-O 5’- GCACTTCGCTCTTCATGG -3’

4118_R-O 5’- TGGGTATACACCATGG -3’

∆ SO4606 (*coxB^-^*)

3471_F-O 5’- CCAGTATTGGCTACAAG -3’

3471_5-O 5’- CGATAATCGTCGTTTAC -3’

3471_5-I 5’- TACTGAGCGGCATGTCACTGTCACAAGACTTCTCC -3’

3471_3-I 5’- CAGTGACATGCCGCTCAGTACATGGAAAGTAGGGG -3’

3471_3-O 5’- GTGGCGCATAGAAGGTCC -3’

3471_R-O 5’- GACTATGGTCACGATCACG -3’

pBBR1MCS5-*aceE* complementation

aceE_F 5’- TTAAGAGAAGGACAGTGTCATGTCTG -3’

aceE_R 5’- GCCTCTTACGCGTACTGTGG -3’

pBBR1MCS5-*aceB* complementation

ACEB_F 5’- CTAAGAGGGCGAGAAGATGAC -3’

ACEB_R 5’- CAATTAAGCAGTTAGCATCTC -3’

pBBR1MCS5-*aceA* complementation

aceA_F 5’- CTAACAGAAAGGAGTGACACTATG -3’

aceA_R 5’- GGCTTAAAACTGCTCTTCTTCG -3’

pBBR1MCS5-*pflB* complementation

PFLB_F 5’- CTAACCAAAAGGTATTAGTACGATG -3’

PFLB _R 5’- GCTTACAGACCTTTTGTGAATG -3’

pBBR1MCS5-*gcvP* complementation

gcvP_F 5’- CATGACCAAGCAAACCCTC -3’

gcvP_R 5’- CCTACTCGTAGTCTGATAACG -3’

pBBR1MCS5-*sucB* complementation

sucB_F 5’- TTAAAAAAGGATCGTTTTTCATGAG -3’

sucB_R 5’- CGTTTATCGAGTTGGATTACAG -3’

pBBR1MCS5-*glyA* complementation

GLYA_F 5’- CTAACCGCTGGAGTAAGCA**ATG**C -3’

GLYA_R 5’- CAGGTTAACCGTAAACAGGG -3’

*^a^*Sequences in italics are added to facilitate fusion of first round PCR amplicons as described by Link (Link et al., 1997).
