# Supplementary material for: Constraint-Based Model of Shewanella oneidensis MR-1 Metabolism: A Tool for Data Analysis and Hypothesis Generation
Source: PLoS Comput Biol. 2010 Jun 24;6(6):e1000822. doi: 10.1371/journal.pcbi.1000822 (PMC2891590; doi:10.1371/journal.pcbi.1000822)
Supplement: Text S1 — Summary of additional experimental results. (0.06 MB DOC) [file pcbi.1000822.s008.doc]

**Pyruvate oxidation.** D and L stereoisomers of lactate are oxidized to pyruvate by two recently found lactate dehydrogenases in MR-1 cells [1]. *S. oneidensis* genome analysis revealed two enzymes dealing with further pyruvate oxidation: pyruvate dehydrogenase complex (PDHc; SO0424-0426) and pyruvate formate-lyase (PFL; SO2912). To determine if there is another enzyme(s) involved in pyruvate oxidation, we constructed two deletion mutants with impaired PDHc (*ΔSO0424*, *aceE^-^*) and PFL (*ΔSO2912*, *pflB^-^*) and tested their ability to grow with pyruvate, lactate, propionate, acetate, and serine under aerobic conditions and on lactate and pyruvate under anaerobic conditions. The *aceE^-^* strain lost the ability to grow with all tested substrates aerobically but retained the ability to use lactate and pyruvate anaerobically. Aerobic growth of *aceE^-^* strain could be achieved on rich media (LB or TSB) only. Conversely, *pflB^-^* strain could not grow anaerobically on lactate or pyruvate, whereas its aerobic growth was not affected (data not shown). Further analysis of culture filtrates showed that lactate was stoichiometrically converted to pyruvate under aerobic conditions by *aceE^-^* strain confirming that there is no other enzyme catalyzing aerobic pyruvate oxidation (Figure S7). Anaerobic incubation of *pflB^-^* strain for 200 hours did not lead to lactate consumption or oxidation, while no fumarate reduction to succinate occurred. These results additionally suggest that the first step of lactate oxidation is accompanied by energy conservation when O_2_ but not fumarate serves as electron acceptor.

**α-Ketoglutarate dehydrogenase.** To test the TCA cycle importance experimentally we tried to construct a deletion mutant (*ΔSO1931*, *sucB^-^*) with impaired α-ketoglutarate dehydrogenase (KGDH) using LB aerobic enrichments. However, this approach appeared to be inappropriate because no colonies could be selected providing the first evidence that an intact TCA cycle is essential for aerobic metabolism of MR-1. Next, selection of a *sucB^-^* strain was attempted using minimal M1 medium supplemented with 10% LB, 20 mM lactate, and 40 mM fumarate under anaerobic conditions. This approach was successful, and *sucB* gene was deleted. Further experiments with *sucB^-^* strain showed that, unlike wild-type culture, it cannot grow aerobically with lactate, pyruvate, acetate, succinate, and LB or TSB, whereas anaerobic growth in M1 medium supplemented with lactate and fumarate was not impaired. Therefore, disruption of TCA cycle at the level of KGDH precluded *S. oneidensis* MR-1 growth with O_2_ as electron acceptor confirming that it is absolutely essential for *S. oneidensis* MR-1 aerobic carbon metabolism inferred from flux balance analysis.

**Serine, glycine, and C1 metabolism.** Serine, glycine, and C1-folate derivatives, particularly 5,10-methylenetetrahydrofolate (methylene-THF), are important precursors of many cell components, including proteins and purines. We investigated the metabolism of these compounds in *S. oneidensis* MR-1 based on computational and experimental approaches. Pathways that can lead to biosynthesis and interconversion of methylene-THF, serine, glycine, and threonine were built based on *S. oneidensis* MR-1 annotated genome analysis (http://www.ncbi.nlm.nih.gov/genomes/lproks.cgi?view=1) and are summarized in Figure S5. It is important to note that methylene-THF, serine, and glycine metabolism are highly intertwined because all enzymes dealing with interconversion of these compounds are bidirectional [2], and each of these compounds can be produced by more than one pathway. Therefore, elucidation of which pathway works under specific experimental conditions (such as carbon source used, growth limitation type etc.) is very important for validation of FBA predictions and overall model improvement.

Serine can be synthesized in *S. oneidensis* from D-3-phosphoglycerate through a canonic three-step pathway [3]. Alternately, serine can be produced from glycine by combining it with C1-unit originated from methylene-THF in reaction catalyzed by serine hydroxymethyltransferase (SHMT) [2,4]. To test the importance of SHMT, we deleted a gene encoding for this enzyme (*SO3471*, *glyA*) and studied growth of the resulting strain on 8 compounds containing from 2 to 6 carbon atoms. Results presented in Figure S6 showed that the mutant lost the ability to grow on propionate, succinate, and α-ketoglutarate, while growth on lactate and serine occurred but only after a long delay (120-240 hours), whereas this mutation just slightly decreased growth rate and cell yield with acetate.

Because FBA predicted that all serine is produced from D-3-phosphoglycerate when *S. oneidensis* uses lactate as the sole source of carbon and energy (Figure 4B), we further investigated growth characteristics of *glyA^-^* mutant on this substrate. Analysis of organic acids in culture liquid revealed that during long lag-phase, lactate oxidation was accompanied by production of acetate and small amount of pyruvate (Figure S2). Characteristically, cultures started to grow only after millimolar concentrations of acetate were accumulated; however, maximal growth rate was about 3-fold slower than that of wild-type or *glyA^-^* cells on acetate. These results demonstrated crucial importance of SHMT for lactate metabolism, while growth on acetate does not involve this enzyme significantly.

To determine which amino acid is produced as the result of SHMT activity we studied the influence of serine and glycine additions on *glyA^-^* strain growth in M1 medium supplemented with lactate. Addition of serine to concentrations between 20-500 mg/l did not produce any changes comparing to control cultures that did not receive this amino acid (data not shown). However, glycine addition produced concentration-dependent growth yield improvement (Figure S3A) while even addition of 10 mg/l of glycine eliminated long lag-phase. Taken together, these results suggest that main function of SHMT during *S. oneidensis* growth on lactate is glycine and, most probably, methylene-THF synthesis. Its function when acetate is the sole source of carbon and energy is unclear, probably it catalyzes production of minor amounts of glycine and methylene-THF based on *glyA^-^* strain slight decrease in growth rate and maximal biomass accumulation comparing to wild-type cultures (between 5 and 10% in three independent experiments).

Besides serine cleavage, there are more pathways that can potentially generate glycine: (1) from threonine (by combined action of threonine dehydrogenase and glycine acetyltransferase or threonine aldolase and acetaldehyde-CoA dehydrogenase), (2) from glyoxylate by means of transamination with alanine or serine, or (3) from formate and CO_2_ (Figure S5). The last pathway cannot be used aerobically because formate in MR-1 cells can be produced from lactate only as the result of pyruvate formate-lyase activity; however, this enzyme is not active in the presence of O_2_ [5]; see also Table 1). Because the remaining two pathways obviously could not complement the function of glycine synthesis in *glyA^-^* strain, it’s unclear whether corresponding genes are not expressed or metabolic regulation prohibits flux from internally made threonine or glyoxylate to glycine.

To determine if threonine pathways can operate in *S. oneidensis*, we tested growth of wild-type and *glyA^-^* strain on lactate in the presence of exogenous threonine (Figure S3B). As in the case with glycine, concentration dependent increase in biomass yield of *glyA^-^* mutant and elimination of long lag-phase was observed, whereas addition of the same concentrations of threonine did not influence growth parameters of wild-type cultures (data not shown). Taken together, these results suggest that exogenous but not internally made threonine can be used as source of glycine by *S. oneidensis*. Finally, inability to use internally produced glyoxylate as a precursor for glycine biosynthesis could be the result of low level of isocitrate lyase activity expressed under aerobic lactate-limited growth (Table 1).

Because synthesis of folate derivatives from internally produced formate cannot work in *S. oneidensis* growing aerobically on lactate (see above), there are only two enzymes encoded in its genome that are able to generate methylene-THF: serine cleavage (catalyzed by SHMT) and glycine cleavage (catalyzed by glycine cleavage system, GCV; SO0779-0781 and SO0426; see Figure S5). A question therefore arises if GCV is important to supply methylene-THF for biosynthetic needs when bacteria grow on lactate and acetate. To address this problem, we deleted a gene (*SO0781*, *gcvP*) encoding for gcvP subunit. The following tests revealed that GCV impairment had not significantly influenced growth *S. oneidensis* on lactate if NH_4_^+^ was used as source of nitrogen (Figure S4A). Alternately, when M1 medium was supplemented with acetate as source of carbon, the mutant grew 2-fold slower than wild-type culture (growth rate was 0.025 h-1 and 0.053 h-1, respectively; Figure S4B). These results in addition to data obtained for *glyA^-^* mutant strongly suggest that GCV does not play important role in production of methylene-THF when lactate serves as the sole source of carbon, whereas *S. oneidensis* growth on acetate significantly depends on GCV activity.

*Shewanella* species are often met in sedimentary eco-niches; particularly, MR-1 strain was isolated from sediments of Oneida Lake, NY [6]. These sediments often contain significant amount of dead biomass’ protein and soluble proteases [7, 8], therefore making amino acids a feasible source of nitrogen for *S. oneidensis*. Our experiments showed that glycine cannot be used as a source of carbon and energy by these bacteria; however, we suggested that another function of GCV may be glycine decomposition in order to produce NH_4_^+^. Experimental results presented in Fig. S6C supported this assumption: while wild-type cells grew with glycine as well as with NH_4_Cl, *gcvP*^-^ mutant showed 2.5-fold decrease in growth rate and produced about 2-fold less biomass before reaching stationary phase. Remaining ability to use glycine as source of nitrogen by the mutant can be attributed to a transaminase activity despite the absence of accordingly annotated gene in *S. oneidensis* genome.

1. Pinchuk GE, Rodionov DA, Yang C, Li X, Osterman AL, et al. (2009) Genomic reconstruction of Shewanella oneidensis MR-1 metabolism reveals a previously uncharacterized machinery for lactate utilization. Proceedings of the National Academy of Sciences 106: 2874-2879.

2. Pasternack LB, Laude DA, Appling DR (1992) Carbon-13 NMR detection of folate-mediated serine and glycine synthesis in vivo in Saccharomyces cerevisiae. Biochemistry 31: 8713-8719.

3. Umbarger HE, Umbarger MA, Siu PML (1963) Biosynthesis of serine in Escherichia coli and Salmonella typhimurium. Journal of Bacteriology 85: 1431–1439.

4. Sauer U, Lasko DR, Fiaux J, Hochuli M, Glaser R, et al. (1999) Metabolic Flux Ratio Analysis of Genetic and Environmental Modulations of Escherichia coli Central Carbon Metabolism. Journal of Bacteriology 181: 6679-6688.

5. Knappe J, Blaschkowski HP, GrÖBner P, Schmitt T (1974) Pyruvate Formate-Lyase of *Escherichia coli*: the Acetyl-Enzyme Intermediate. European Journal of Biochemistry 50: 253-263.

6. Myers CR, Nealson KH (1988) Bacterial manganese reduction and growth with manganese oxide as the sole electron acceptor. Science 240: 1319-1321.

7. Lochte K, Boetius A, Petry C (2000) Microbial food webs under severe nutrient limitations: life in the deep sea. Bell, CR, Brylinsky, M, Johnson-Green, P (eds) Microbial Biosystems: New Frontiers Proceedings of the 8th International Symposium on Microbial Ecology Atlantic Canada Society for Microbial Ecologgy, Halifax, Canada: 95-102.

8. Danovaro R, Armeni M, Dell'Anno A, Fabiano M, Manini E, et al. (2001) Small-Scale Distribution of Bacteria, Enzymatic Activities, and Organic Matter in Coastal Sediments. Microbial Ecology 42: 177-185.
