# Supplementary material for: Constraint-Based Model of Shewanella oneidensis MR-1 Metabolism: A Tool for Data Analysis and Hypothesis Generation
Source: PLoS Comput Biol. 2010 Jun 24;6(6):e1000822. doi: 10.1371/journal.pcbi.1000822 (PMC2891590; doi:10.1371/journal.pcbi.1000822)
Supplement: Text S2 — Supplemental experimental methods. (0.05 MB DOC) [file pcbi.1000822.s009.doc]

**Lipid extraction, weighing, separation, fatty acid analysis, and ESI-MS/MS lipid profling:**

A known volume of cultural liquid was frozen at -20^o^C and then lyophilized. Lyophilized samples were transferred to glass tubes and extracted by the method of Bligh and Dyer (1959). With the samples in pre-weighed vials, the solvent was evaporated under nitrogen, then under vacuum, and the samples, were weighed to determine lipid weight by subtraction of the vial weight. The samples were dissolved in 1.0 ml chloroform and 0.03 to 0.12 ml applied to an Analtech CN SPE 500mg packing column. Neutral lipids were eluted with 10 ml hexane/ether/acetic acid (80/20/1) and polar lipids were eluted with 10 ml chloroform/methanol/water (4/1/0.1). Pentadecanoic acid was added to the polar lipid fraction as an internal standard. The fatty acid methyl esters were formed by reacting the lipid with 1.5 N methanolic HCl (Supelco) for 30 min at 78ºC. Fatty acid methyl esters were separated by gas chromatography using a temperature gradient of 100ºC for 2 min, ramp 4ºC per min to 250ºC , hold at 250ºC for 10 min on a Supelco 30 meter SP2380 capillary column and detected by flame ionization.

An automated electrospray ionization-tandem mass spectrometry approach was used for lipid profiling, and data acquisition and analysis and acyl group identification were carried out as described previously (Devaiah et al., 2006) with modifications. The samples were dissolved in 1 ml chloroform. An aliquot of 3 to 10 µl of extract in chloroform was used. Precise amounts of internal standards, obtained and quantified as previously described (Welti et al., 2002), were added in the following quantities (with some small variation in amounts in different batches of internal standards): 0.30 nmol di12:0-PE, 0.30 nmol di23:0-PE, 0.30 nmol 14:0-lysoPE, 0.30 nmol 18:0-lysoPE, 0.30 nmol di14:0-PG, 0.30 nmol di20:0(phytanoyl)-PG, 0.30 nmol 14:0-lysoPG, 0.30 nmol 18:0-lysoPG, 0.30 nmol di14:0-PA, and 0.30 nmol di20:0(phytanoyl)-PA. The sample and internal standard mixture was combined with solvents, such that the ratio of chloroform/methanol/300 mM ammonium acetate in water was 300/665/35, and the final volume was 1.2 ml.

The polar lipid fractions were introduced by continuous infusion into the ESI source on a triple quadrupole MS/MS (4000 QTrap, Applied Biosystems, Foster City, CA). Samples were introduced using an autosampler (LC Mini PAL, CTC Analytics AG, Zwingen, Switzerland) fitted with the required injection loop for the acquisition time and presented to the ESI needle at 30 μl/min.

Sequential precursor and neutral loss scans of the extracts produce a series of spectra with each spectrum revealing a set of lipid species containing a common head group fragment. Lipid species were detected with the following scans: PE and lysoPE, [M + H]^+^ ions in positive ion mode with Neutral Loss of 141.0 (NL 141.0); PG, [M + NH4]^+^ in positive ion mode with NL 189.0 for PG; lysoPG, [M – H]^-^ in negative mode with Pre 152.9; and PA, [M + NH4]^+^ in positive ion mode with NL 115.0. The scan speed was 50 or 100 u per sec. The collision gas pressure was set at 2 (arbitrary units). The collision energies, with nitrogen in the collision cell, were +28 V for PE and lysoPE, +25 V for PA, -57 V for lysoPG, and +20 V for PG. Declustering potentials were +100 V or -100 V. Entrance potentials were +15 V for PE and lysoPE, -10 V for lysoPG, and +14 V for PA and PG. Exit potentials were +11 V for PE and lysoPE, -14 V for lysoPG, and +14 V for PA and PG. The mass analyzers were adjusted to a resolution of 0.7 u full width at half height. For each spectrum, 9 to 150 continuum scans were averaged in multiple channel analyzer (MCA) mode. The source temperature (heated nebulizer) was 100 °C, the interface heater was on, +5.5 kV or -4.5 kV were applied to the electrospray capillary, the curtain gas was set at 20 (arbitrary units), and the two ion source gases were set at 45 (arbitrary units).

The background of each spectrum was subtracted, the data were smoothed, and peak areas integrated using a custom script and Applied Biosystems Analyst software. The lipids in each class were quantified in comparison to the two internal standards of that class. A set of mass spectra was acquired on the internal standard mixture only, and peaks corresponding to the target lipids in these spectra were identified and molar amounts calculated in comparison to the internal standards on the same lipid class. To correct for chemical or instrumental noise in the samples, the molar amount of each lipid metabolite detected in the “internal standards only” spectra was subtracted from the molar amount of each metabolite calculated in each sample spectrum. Finally, the data were corrected for the fraction of the sample analyzed and normalized to the sample lipid weights to produce data in the units nmol/mg.

Bligh,E.G., and Dyer,W.J. 1959. A rapid method for total lipid extraction and purification. Can.J.Biochem.Physiol. 37, 911-917.

Devaiah, S. P., Roth, M. R., Baughman, E., Li, M., Tamura, P., Jeannotte, R., Welti, R., and Wang, X. 2006. Quantitative profiling of polar glycerolipid species and the role of phospholipase Dα1 in defining the lipid species in Arabidopsis tissues.  Phytochemistry 67, 1907-1924.

Welti, R., Li, W., Li, M., Sang, Y., Biesiada, H., Zhou, H., Rajashekar, C.B., Williams, T.D., and Wang, X.  2002.  Profiling membrane lipids in plant stress responses: Role of phospholipase Dα in freezing-induced lipid changes in Arabidopsis. J. Biol. Chem. 277, 31994-32002.

**Amino acid composition of biomass.**

To analyze amino acid composition of biomass protein, cells were harvested (4,000 x g, 10 min., 4°C) and washed twice with 0.9% NaCl. Fifty μg of cells were dried under vacuum (Eldex hydrolysis/derivatization station, Eldex laboratories, Inc., Napa, CA, USA), hydrolized with 6N HCl containing 1% of phenol at 116°C for 24 h, and resuspended in 100 μl of 20 mM HCl containing 0.05 mM of α-aminobutyric acid (AAba) as an internal standard. The amino acids present in 20 μl of a sample were derivatized with AccQ-Fluor reagent (Waters Corp., Milford, MA, USA) at 55°C for 10 min according to the manufacture instructions and separated by reversed-phase high-performance liquid chromatography (HPLC) as follows: 5 μl of the sample was injected into a 4-μm AccQ-Tag Nova-Pak C-18 column (3.9 mm x 150 mm, Waters Corp., Milford, MA, USA), eluted using a linear gradient of acetonitrile (from 1.2% to 4.2% over 15 min, from 4.2% to 6% over 4 min, from 6% to 20% over 12 min, at 20% over 1 min, from 20% to 60% over 1 min) with a flow rate of 1.0 ml/min at 37°C, and detected at 254 nm (HPLC system and UV detector by Shimadzu, Tokyo, Japan). The amino acids in the samples were determined and quantified comparing the peak areas eluted from the column with those of 50 pmol amino acids contained in a standard mix (Sigma Aldrich Inc., St. Louis, MO). AAba (0.05 mM) was also added to the standard mix as internal control to compare also the derivatization efficiency. The data were normalized to the dried organic cell weight.

**Preparation of Crude Extracts**

Cells were harvested by centrifugation for 15 minutes at 8^o^C and 10,000 x g and then washed three times in 50 mM Tris-HCl (pH=7.5). Crude cellular extracts were prepared by homogenization in a bead beater for 5 minutes at 4^o^C or by sonication at 4^o^C. The lysates were then clarified by centrifugation at 4^o^C and 20,000 x g for 30 minutes to form the cell-free extracts for each assay. Protein in crude extracts was quantified using a BCA protein assay kit (Pierce, Rockford, IL). All assays conditions were preliminary optimized.

**Enzymes activity measurements**

All assays were done in a final volume of 1 ml at 30^o^C by using a Shimadzu UV-2101PC scanning spectrophotometer equipped with a thermostat (Shimadzu Scientific Instruments, Columbia, MD). Formation/consumption of NAD(P)^+^ was monitored and recorded at 340 nm (extinction coefficient=6.22 mM^–1^ cm^–1^), 5-Thio-2-Nitrobenzoic acid at 412 nm (extinction coefficient=13.6 mM^–1^ cm^–1^), and phenylhydrazine-glyoxylate complex at 324 nm (extinction coefficient=16.8 mM^–1^ cm^–1^). One unit of enzyme activity was defined as the amount of enzyme that produced 1 µmol of product per minute. Crude extracts were added to assay mixtures to make final concentration between 0.1 and 1 mg/ml. The assay conditions used were as follows:

- Pyruvate dehydrogenase activity was measured by following formation of NADH in the presence of 50 mM Tris-HCl (pH=7.8), 2.5 mM NAD^+^, 0.2 mM thiamin pyrophosphate, 0.1 mM coenzyme A, 0.3 mM dithithreitol, 5 mM pyruvate, and 1 mM magnesium chloride. The reaction was initiated by addition of pyruvate.
- Pyruvate-formate lyase activity was determined by following the reduction of NAD^+^ in an air-tight anaerobic cuvette with silicone septum seal. The assay mixture contained 50 mM Tris-HCl (pH=8.0), 2.0 units of citrate synthase (porcine heart, EC 4.1.3.7), 30 units of malate dehydrogenase (porcine heart, EC 1.1.1.37), 10 mM dithiothreitol, 20 mM sodium pyruvate, 10 mM DL-sodium malate, 0.055 mM coenzyme A, and between 1 to 3 mM NAD^+^. The reaction was initiated by the injection of freshly made crude extract prepared in the presence of 10 mM DTT.
- NAD^+^ or NADP^+^-coupled isocitrate dehydrogenase activity was measured by following formation of NADH or NAD(P)H in the presence of 50 mM Tris-HCl (pH=8.0), 2.5 mM magnesium chloride, 2 mM NAD^+^ or NADP^+^, 2 mM isocitrate, and 0.5-1 mg/ml of crude extract protein. The reaction was initiated by addition of isocitrate.
- Malate dehydrogenase activity was determined by measuring formation of NAD^+^ in the presence of 50 mM Tris-HCl (pH=7.2), 0.2 mM NADH, and 2.0 mM oxaloacetate. The reaction was initiated by addition of oxaloacetate.
- Malate synthase activity was determined spectrophotometrically by measuring formation of 5-thio-2-nitrobenzoic acid at 412 nm in the presence of 30 mM Tris-HCl buffer (pH=8.0), 10 mM magnesium chloride, 0.25 mM acetyl-CoA, 1 mM glyoxylic acid, and 0.2 mM 5,5'-dithio-bis(2-nitrobenzoic acid). The reaction was initiated by addition of glyoxylic acid.
- Isocitrate lyase activity was determined by a standard spectrophotometric method that measures the formation phenylhydrazine-glyoxylate complex at 324 nm in the presence of 50 mM Tris-HCl (pH=6.8), 5 mM magnesium chloride, 1 mM isocitrate, and 1 mM ethylenediaminetetraacetic acid. The reaction was initiated by addition of isocitrate.
- Hydroxypyruvate reductase activity was determined by following the formation of NAD^+^ in the presence of 50 mM Tris-HCl (pH=6.6), 0.2 mM NADH, and 3.5 mM lithium hydroxypyruvate. The reaction was initiated by addition hydroxypyruvate.
