# Supplementary material for: Constraint-Based Model of Shewanella oneidensis MR-1 Metabolism: A Tool for Data Analysis and Hypothesis Generation
Source: PLoS Comput Biol. 2010 Jun 24;6(6):e1000822. doi: 10.1371/journal.pcbi.1000822 (PMC2891590; doi:10.1371/journal.pcbi.1000822)
Supplement: Text S3 — Methods for cycle calculations. (0.13 MB DOC) [file pcbi.1000822.s010.doc]

*Enumeration of Cycles*

We developed an optimization-based approach to calculate cycles in constraint-based models of metabolism. In both cases described below, all the exchange fluxes are constrained to be zero so that no metabolites can enter or exit the system. To find cycles where a net transformation takes place a backwards reaction must be added to the network that cancels out the cycle’s net transformation. For example, for ATP-dependent futile cycles the net transformation of the cycle is ATP + H_2_O 🡪 ADP + P_i_ + H^+^, so a backwards reaction (ATPS) needs to be added that does the opposite reaction (i.e. ADP + P_i_ + H^+^ 🡪 ATP + H_2_O). By constraining a positive flux through this backwards reaction, a cycle that does the forward reaction will have to carry a non-zero flux to counteract the flux through the backwards reaction.

The identification of cycles can be used as an initial evaluation of the reconstructed network and can be used to identify problematic reactions in the network as described below. Cycles where the net transformation is a transhydrogenase reaction can also be identified by adding the backwards transhydrogenase reaction to the network (if not already present). Some organisms lack transhydrogenase enzymes and must balance the NAD(H) and NADP(H) pools separately, so these cycles would need to be eliminated by removing reactions or changing co-factor usage in the reactions. Cycles that result in the free production of ATP also should be eliminated from all networks and they can be found by using a backwards reaction that hydrolyzes ATP (ATP + H_2_O 🡪 ADP + P_i_ + H^+^).

A mixed-integer linear programming problem can be formulated (Eq. 1-6) to minimize the number of reactions included in the cycle (Eq. 1), where *y_j_* is a decision variable that indicates whether a flux is zero (*y_j_*=0) or non-zero (*y_j_*=1). If the flux through the reaction is non-zero the corresponding value of the decision variable, *y_j_*, must be one (Eq. 3). The fluxes and decision variables must satisfy the mass balance (Eq. 2), thermodynamic, enzyme capacity constraints (Eq. 3), and a non-zero flux constraint through the cycle’s backwards reaction (i) (Eq. 4, where ε is a positive number). The total number of reactions is *n*.

(Eq. 1)

(Eq. 2)

(Eq. 3)

$v_{i}>e$ where *i* is the cycle’s backwards reaction (Eq. 4)

for all *k* previous iterations (Eq. 5)

(Eq. 6)

To find additional cycles, integer-cut constraints can be added (Eq. 5) which ensure that the same solution is not revisited and that a new solution is not a combination of previous solutions [1], where *y_j_^k^* indicates whether reaction *j* was used in the previous iteration *k*. As more cycles are found the number of integer-cut constraints increases, so for the first solution there will be no integer-cut constraints and for the m^th^ solution there will be m-1 integer-cut constraints (with *k*=1…m-1)

Circulations are cycles with no net transformation [2] and a net flux through these circulations is thermodynamically infeasible [3,4]. These circulations can also be identified by replacing Eq. 4 with constraints requiring non-zero fluxes through at least one reaction in the network (Eq. 13). The problem needs to be reformulated to turn it into a mixed-integer linear programming problem and consists of Eqs. 7-14.

(Eq. 7)

(Eq. 8)

(Eq. 9)

(Eq. 10)

(Eq. 11)

$x_{j}+y_{j}\leq1$ for all j reactions (Eq. 12)

for all j reactions (Eq. 13)

for all k previous iterations (Eq. 14)

(Eq. 15)

In this problem the flux through the original reaction (*v_j_*) is decomposed into its forward (*v_j_^for^*) and reverse fluxes (*v_j_^rev^*) using Eq. 9. Both the forward and reverse fluxes are constrained to be non-negative (Eq. 10-11). The upper limit of the forward flux is constrained by Eq. 10 using *x_j_*, the decision variable indicating whether the forward reaction is included in the cycle, and the upper limit of the flux through the original reaction (*β_j_* which has the same value as in Eq. 3). The upper limit of the reverse flux is constrained by Eq. 11 using *y_j_*, the decision variable indicating whether the reverse reaction is included in the cycle, and the negative of the lower limit of the flux through the original reaction (α*_j_* which has the same value as in Eq. 3). So if a reaction is irreversible then *α_j_* = 0 and *v_j_^rev^* = 0. To ensure that the forward and reverse fluxes do not carry flux simultaneously Eq. 12 is added so that if the forward flux is non-zero (*x_j_*=1) then the reverse flux has to be zero (*y_j_*=0). To ensure a circulation is found, a constraint is added the sum of the forward and reverse fluxes have to be greater than ε (a positive number). Integer-cut constraints (Eq. 14) can be added to find additional cycles ensuring that previous ones are not revisited. Cycles that contain the minimum number of non-zero forward and reverse reactions are found first and each subsequent cycle must not use at least one original reaction from each of the previous solutions.

**References:**

1. Burgard AP, Vaidyaraman S, Maranas CD (2001) Minimal reaction sets for Escherichia coli metabolism under different growth requirements and uptake environments. Biotechnol Prog 17: 791-797.

2. Teusink B, Wiersma A, Molenaar D, Francke C, de Vos WM, et al. (2006) Analysis of growth of Lactobacillus plantarum WCFS1 on a complex medium using a genome-scale metabolic model. J Biol Chem 281: 40041-40048.

3. Price ND, Famili I, Beard DA, Palsson BO (2002) Extreme pathways and Kirchhoff's second law. Biophys J 83: 2879-2882.

4. Beard DA, Liang SD, Qian H (2002) Energy balance for analysis of complex metabolic networks. Biophys J 83: 79-86.

Below is an example network and the corresponding futile cycle and circulations that are identified using the above approach. The GAMS code is also provided below to solve for the cycles in this network. This code can be run on the demo version of GAMS which can be downloaded for free from the GAMS website (www.gams.com).


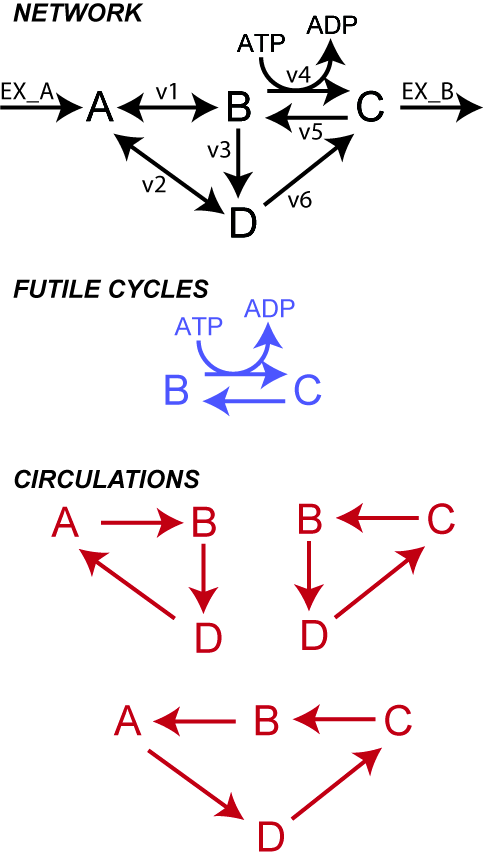


**GAMS CODE:**

$onecho > cplex.opt

eprhs 1e-8

epopt 1e-8

epint 1e-8

$offecho

Set i /A,B,C,D,ATP,ADP/

j /v1,v2,v3,v4,v5,v6,EX_A,EX_C,ATPS/

store /n1*n50/

exch(j) /EX_A,EX_C/;

alias(temp,store);

Parameter LowerLimits(j) /(v1,v2) -100/

UpperLimits(j);

Table S(i,j)

v1 v2 v3 v4 v5 v6 EX_A EX_C ATPS

A -1 -1 0 0 0 0 1 0 0

B 1 0 -1 -1 1 0 0 0 0

C 0 0 0 1 -1 1 0 -1 0

D 0 1 1 0 0 -1 0 0 0

ATP 0 0 0 -1 0 0 0 0 1

ADP 0 0 0 1 0 0 0 0 -1 ;

UpperLimits(j)=100;

UpperLimits(exch)=0;

LowerLimits(exch)=0;

Parameters PreviousY(j,store),PreviousObj(store),counter;

Variables

v(j) flux values through reaction in network

Obj objective function;

Positive Variables vfor(j),vrev(j);

Integer Variables y(j),x(j);

Equations

massbalance(i) mass balance equations for each metabolite

futile_obj

upperbounds(j)

lowerbounds(j)

integercuts(store) prevents same reactions from being used in future solutions

massbalance_circulation(i) mass balance equations for each metabolite

circulation_obj

minflux

pickone(j)

upperbounds_for(j)

upperbounds_rev(j)

integercuts_circulation(store) prevents same reactions from being used in future solutions;

massbalance(i).. sum( j,S(i,j)*v(j) )=e=0;

futile_obj.. Obj=e=sum(j, y(j) );

upperbounds(j).. v(j)=l=UpperLimits(j)*y(j);

lowerbounds(j).. v(j)=g=LowerLimits(j)*y(j);

integercuts(store).. sum(j, PreviousY(j,store)*(y(j)) )=l= -1+PreviousObj(store);

massbalance_circulation(i).. sum(j,S(i,j)*(vfor(j)-vrev(j)) )=e=0;

circulation_obj.. Obj=e=sum(j, y(j)+x(j) );

minflux.. sum(j, vfor(j)+vrev(j) )=g=1;

pickone(j).. x(j)+y(j)=l=1;

upperbounds_for(j).. vfor(j)=l=UpperLimits(j)*y(j);

upperbounds_rev(j).. vrev(j)=l=-LowerLimits(j)*x(j);

integercuts_circulation(store).. sum(j, PreviousY(j,store)*(y(j)+x(j)) )=l= -1+PreviousObj(store);

Model Futile /massbalance,futile_obj,integercuts,upperbounds,lowerbounds/;

option mip=cplex;

Futile.optfile=1;

Model Circulations /massbalance_circulation,circulation_obj,minflux,pickone,upperbounds_for,upperbounds_rev,integercuts_circulation/;

Circulations.optfile=1;

file results /Cycles_Example.txt/;

results.pw=5000; results.ap=1; results.pc=5; results.ps=130;

put results;

put "Futile Cycles"; put /;

putclose results;

*CALCULATE FUTILE CYCLES

PreviousY(j,store)=0; PreviousObj(store)=2000; counter=1;

v.fx('ATPS')=1; LowerLimits('ATPS')=0;

loop(temp,

if(counter<2000,

solve Futile using mip minimizing Obj;

if (Futile.ModelStat=1,

PreviousY(j,temp)=y.l(j);

PreviousObj(temp)=Obj.l;

put results;

loop(j, if(y.l(j)>1e-8, put j.tl));

put /;

putclose results; );

counter=PreviousObj(temp); );

);

put results; put/;

put "Circulations"; put /;

putclose results;

*CALCULATE CIRCULATIONS

LowerLimits('ATPS')=0; UpperLimits('ATPS')=0;

PreviousY(j,store)=0; PreviousObj(store)=2000; counter=1;

loop(temp,

if(counter<2000,

solve Circulations using mip minimizing Obj;

if (Circulations.ModelStat=1,

PreviousY(j,temp)=y.l(j)+x.l(j);

PreviousObj(temp)=Obj.l;

put results;

loop(j, if(y.l(j)+x.l(j)>1e-8, put j.tl));

put /;

putclose results; );

counter=PreviousObj(temp); );

);
