# Supplementary material for: Constraint-Based Model of Shewanella oneidensis MR-1 Metabolism: A Tool for Data Analysis and Hypothesis Generation
Source: PLoS Comput Biol. 2010 Jun 24;6(6):e1000822. doi: 10.1371/journal.pcbi.1000822 (PMC2891590; doi:10.1371/journal.pcbi.1000822)
Supplement: Figure S1 — Growth dynamics of S. oneidensis MR-1 wild-type (filled circles), and selected deletion mutants. Panel A: ΔSO3855 (open triangle) and ΔSO4118 (open diamond). Panel B: ΔSO4606 (open circle) and (filled triangle) double mutant ΔSO4606/SO2363. Cells were cultivated in 100-well plates in Bioscreen C; each well (550 µl total volume) received 100 µl of M1 medium supplemented with 20 mM D,L-lactate. (0.10 MB PDF) [file pcbi.1000822.s011.pdf]

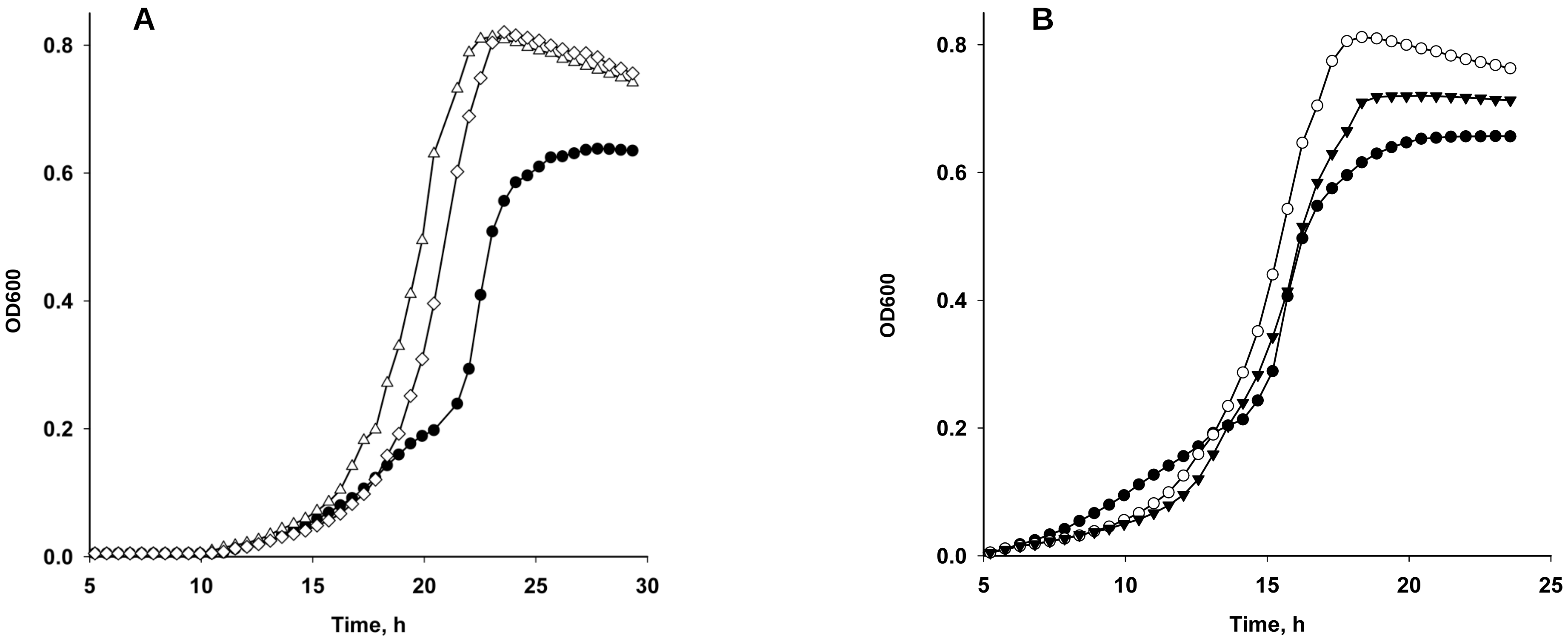

Figure S1. Growth dynamics of *S. oneidensis* MR-1 wild-type (●), and selected deletion mutants. Panel A: *ΔSO3855* (▵) and *ΔSO4118* (◊). Panel B: *ΔSO4606* (○) and (▼) double mutant *ΔSO4606/SO2363*. Cells were cultivated in 100-well plates in Bioscreen C; each well (550 µl total volume) received 100 µl of M1 medium supplemented with 20 mM D,L-lactate.
