# Supplementary material for: Constraint-Based Model of Shewanella oneidensis MR-1 Metabolism: A Tool for Data Analysis and Hypothesis Generation
Source: PLoS Comput Biol. 2010 Jun 24;6(6):e1000822. doi: 10.1371/journal.pcbi.1000822 (PMC2891590; doi:10.1371/journal.pcbi.1000822)
Supplement: Figure S2 — Growth of S. oneidensis MR-1 ΔSO3471 mutants on lactate (45 mM) in M1 medium and organic acids concentration dynamics. Cells were added in the beginning to make final optical density 0.08 at 600 nm. Crimp-sealed serum bottles were used for cultivation. (0.03 MB PDF) [file pcbi.1000822.s012.pdf]

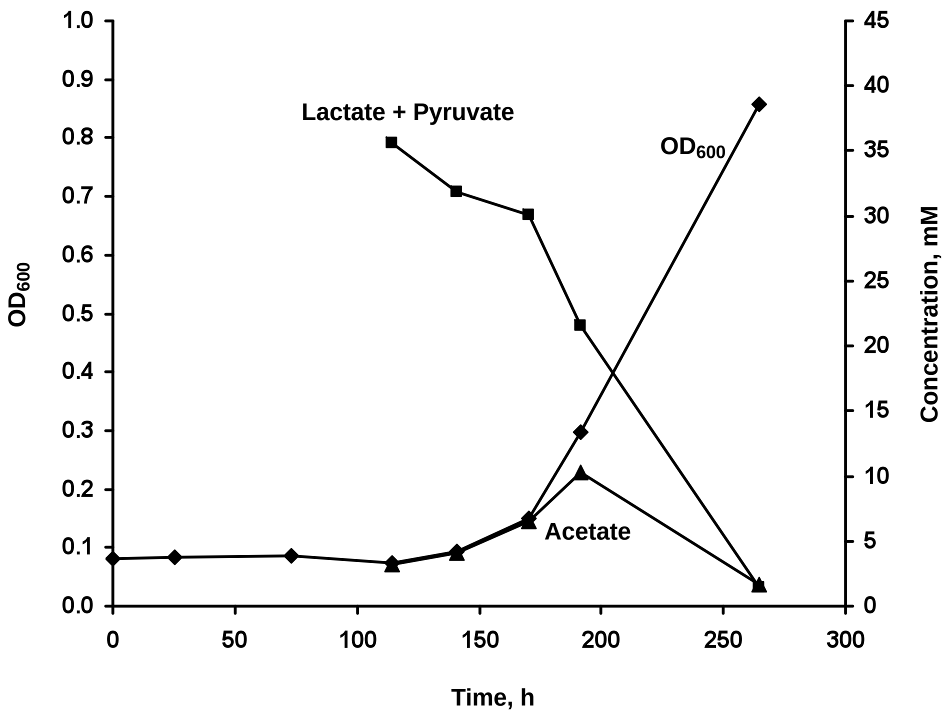

Figure S2. Growth of *S. oneidensis* MR-1 *ΔSO3471* mutants on lactate (45 mM) in M1 medium and organic acids concentration dynamics. Cells were added in the beginning to make final optical density 0.08 at 600 nm. Crimp-sealed serum bottles were used for cultivation
