# Supplementary material for: Constraint-Based Model of Shewanella oneidensis MR-1 Metabolism: A Tool for Data Analysis and Hypothesis Generation
Source: PLoS Comput Biol. 2010 Jun 24;6(6):e1000822. doi: 10.1371/journal.pcbi.1000822 (PMC2891590; doi:10.1371/journal.pcbi.1000822)
Supplement: Figure S3 — Influence of glycine (A) and threonine (B) additions on maximal accumulation of S. oneidensis MR-1 ΔSO3471 biomass on lactate (45 mM) in M1 medium. Biomass accumulation was expressed as optical density at 600 mn. Crimpsealed serum bottles were used for cultivation. (0.12 MB PDF) [file pcbi.1000822.s013.pdf]

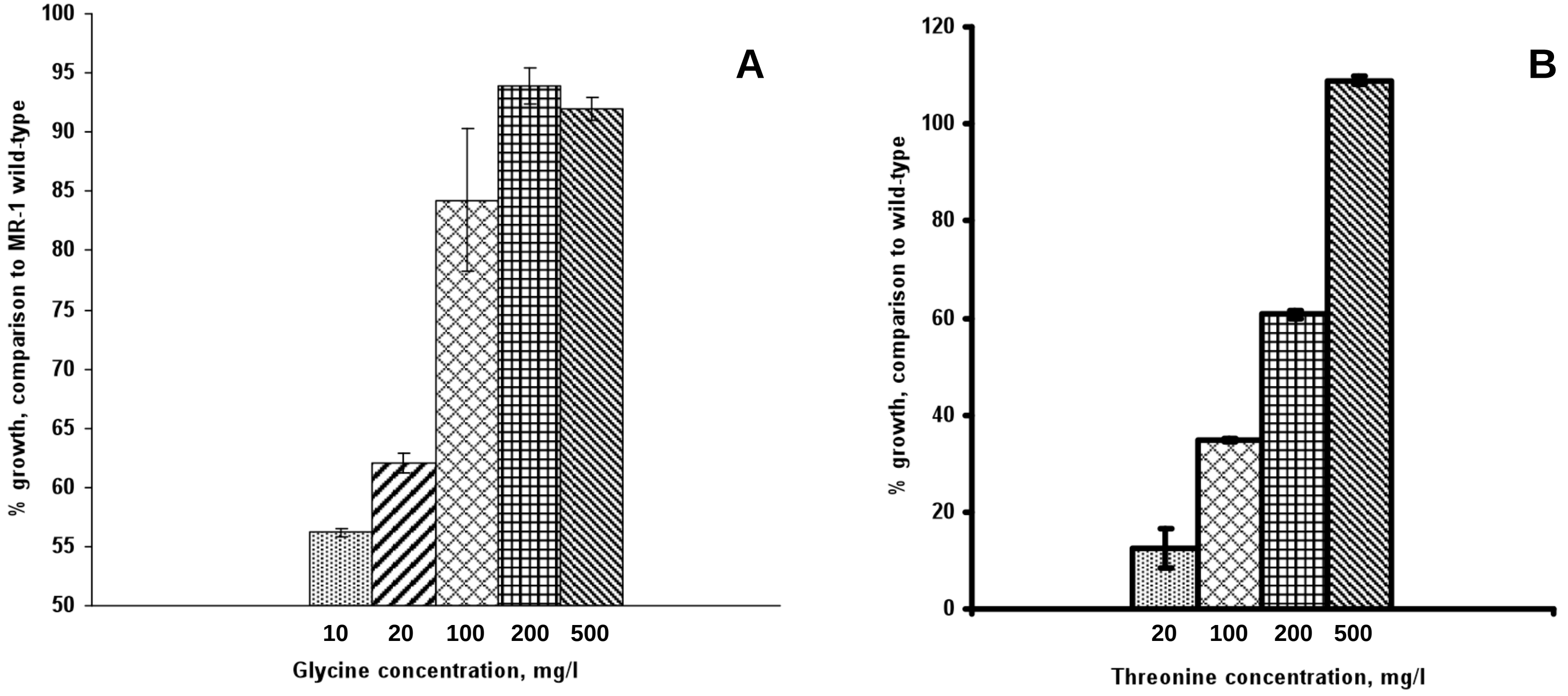

Figure S3. Influence of glycine (A) and threonine (B) additions on maximal accumulation of *S. oneidensis* MR-1 *ΔSO3471* biomass on lactate (45 mM) in M1 medium. Biomass accumulation was expressed as optical density at 600 mn. Crimp-sealed serum bottles were used for cultivation.
