# Supplementary material for: Constraint-Based Model of Shewanella oneidensis MR-1 Metabolism: A Tool for Data Analysis and Hypothesis Generation
Source: PLoS Comput Biol. 2010 Jun 24;6(6):e1000822. doi: 10.1371/journal.pcbi.1000822 (PMC2891590; doi:10.1371/journal.pcbi.1000822)
Supplement: Figure S4 — Growth dynamics of S. oneidensis MR-1 wild-type and selected deletion mutants. Wild-type (filled circle) and ΔSO0781 (open circle) deletion mutant were grown on lactate (18 mM) or acetate (45 mM) in M1 medium supplemented with NH4Cl or glycine (10 mM) as the sole source of nitrogen. Crimp-sealed serum bottles were used for cultivation, starting OD600 values were 0.01 (A and B) and 0.003 (C). (0.08 MB PDF) [file pcbi.1000822.s014.pdf]

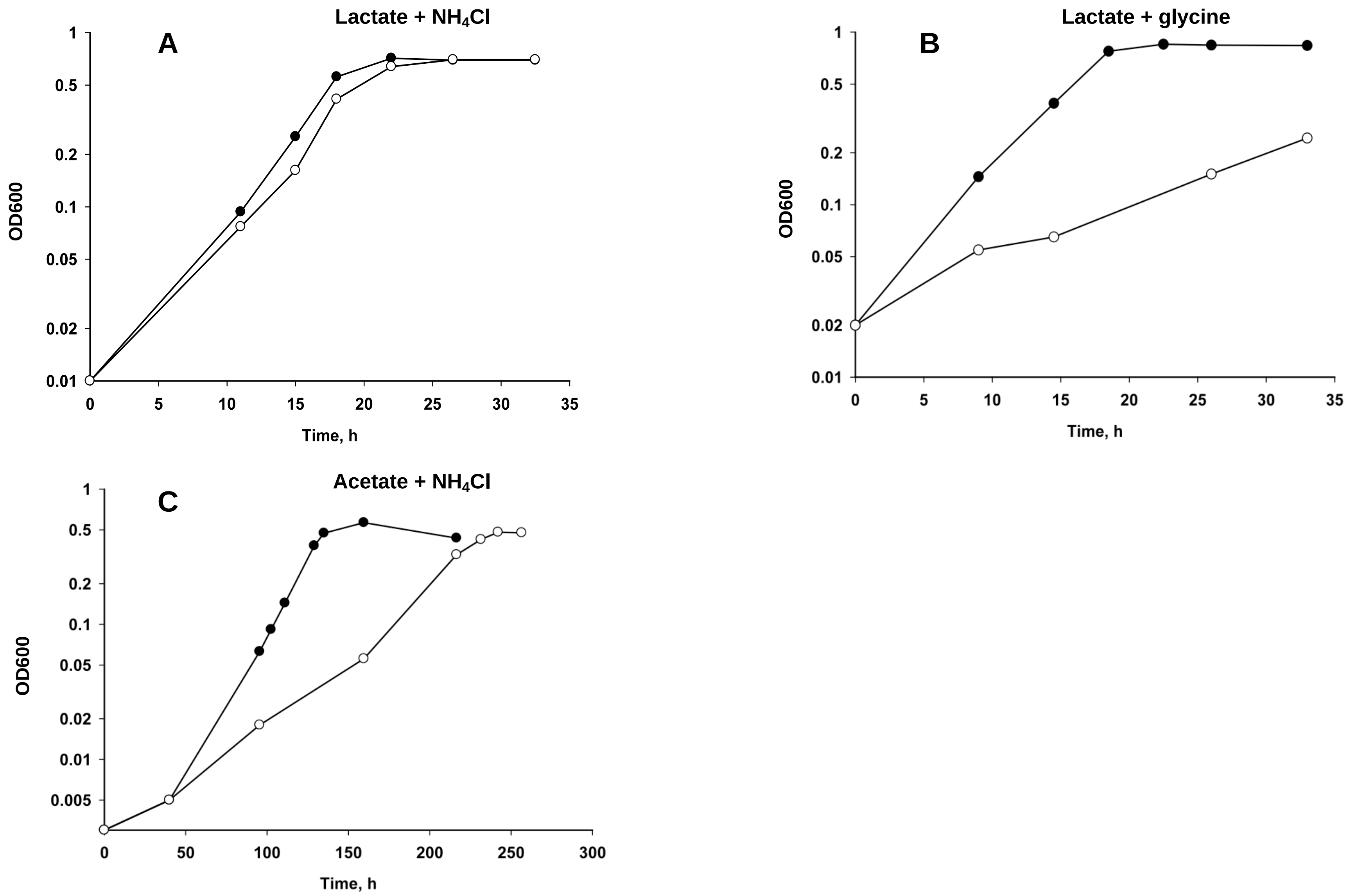

Figure S4. Growth dynamics of *S. oneidensis* MR-1 wild-type and selected deletion mutants. Wild-type (●) and *ΔSO0781* (○) deletion mutant were grown on lactate (18 mM) or acetate (45 mM) in M1 medium supplemented with NH_4Cl or glycine (10 mM) as the sole source of nitrogen. Crimp-sealed serum bottles were used for cultivation, starting OD_{600} values were 0.01 (A and B) and 0.003 (C).
