# Supplementary material for: Constraint-Based Model of Shewanella oneidensis MR-1 Metabolism: A Tool for Data Analysis and Hypothesis Generation
Source: PLoS Comput Biol. 2010 Jun 24;6(6):e1000822. doi: 10.1371/journal.pcbi.1000822 (PMC2891590; doi:10.1371/journal.pcbi.1000822)
Supplement: Figure S5 — Metabolic network for interconversion of C1-compounds and biosynthesis/degradation of relevant amino acids in S. oneidensis MR-1. The network was constructed based on genome annotation. (0.10 MB PDF) [file pcbi.1000822.s015.pdf]

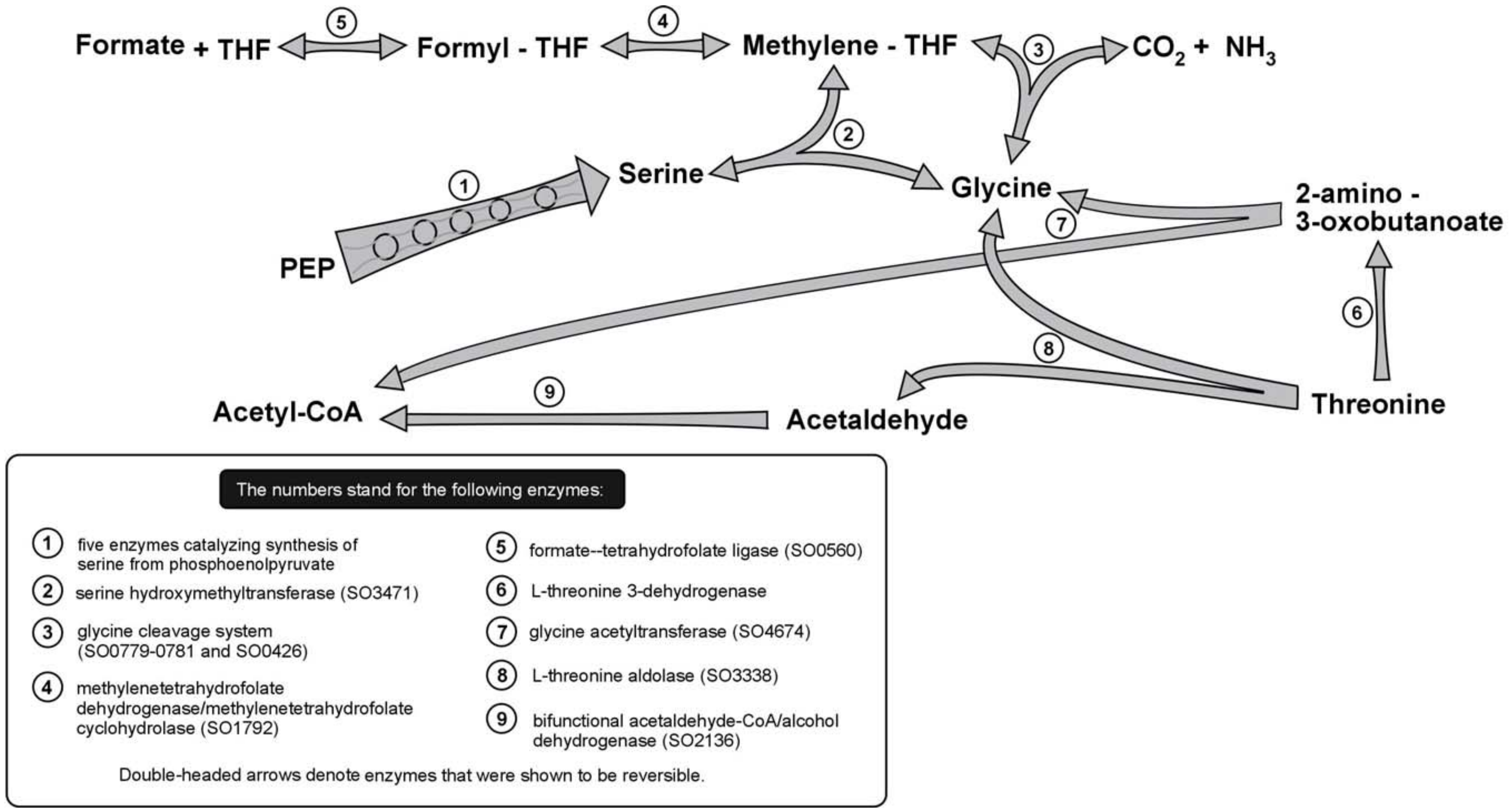

Figure S5. Metabolic network for interconversion of C1-compounds and biosynthesis/degradation of relevant amino acids in *S. oneidensis* MR-1. The network was constructed based on genome annotation.
