# Supplementary material for: Constraint-Based Model of Shewanella oneidensis MR-1 Metabolism: A Tool for Data Analysis and Hypothesis Generation
Source: PLoS Comput Biol. 2010 Jun 24;6(6):e1000822. doi: 10.1371/journal.pcbi.1000822 (PMC2891590; doi:10.1371/journal.pcbi.1000822)
Supplement: Figure S6 — Growth of S. oneidensis MR-1 wild-type (A) and ΔSO3471 mutant (B) in M1 medium supplemented with different compounds as sole sources of carbon and energy. Crimp-sealed serum bottles were used for cultivation, and starting OD600 value was 0.015 for all experiments. (0.13 MB PDF) [file pcbi.1000822.s016.pdf]

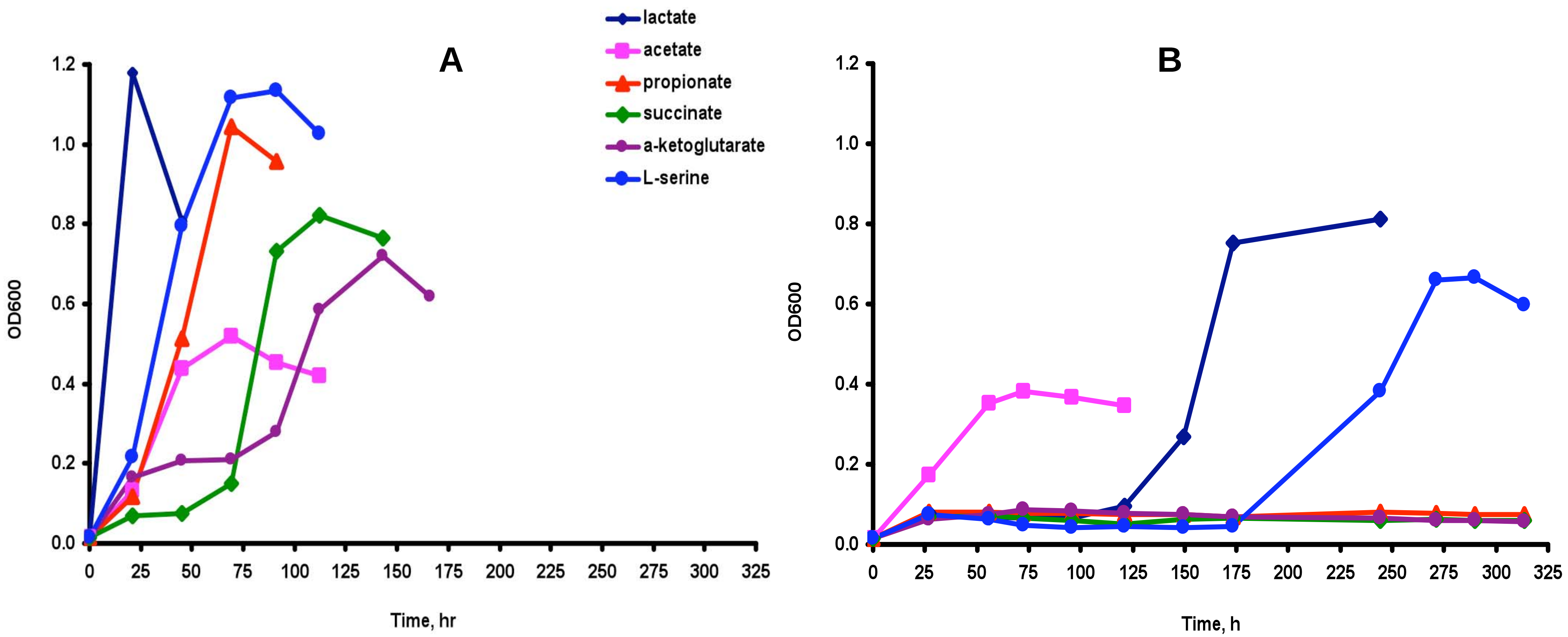

Figure S6. Growth of *S. oneidensis* MR-1 wild-type (A) and *ΔSO3471* mutant (B) in M1 medium supplemented with different compounds as sole sources of carbon and energy. Crimp-sealed serum bottles were used for cultivation, and starting OD_{600} value was 0.015 for all experiments.
