# Supplementary material for: Constraint-Based Model of Shewanella oneidensis MR-1 Metabolism: A Tool for Data Analysis and Hypothesis Generation
Source: PLoS Comput Biol. 2010 Jun 24;6(6):e1000822. doi: 10.1371/journal.pcbi.1000822 (PMC2891590; doi:10.1371/journal.pcbi.1000822)
Supplement: Figure S7 — Aerobic growth of S. oneidensis ΔSO0424 cells in M1 medium supplemented with 18 mM D,L-lactate. 70 ml serum bottles containing 15 ml of medium were used. Periodically 0.7–0.8 ml samples were withdrawn to measure OD600 and organic acids in 0.22 µm filtrates of culture. (0.07 MB PDF) [file pcbi.1000822.s017.pdf]

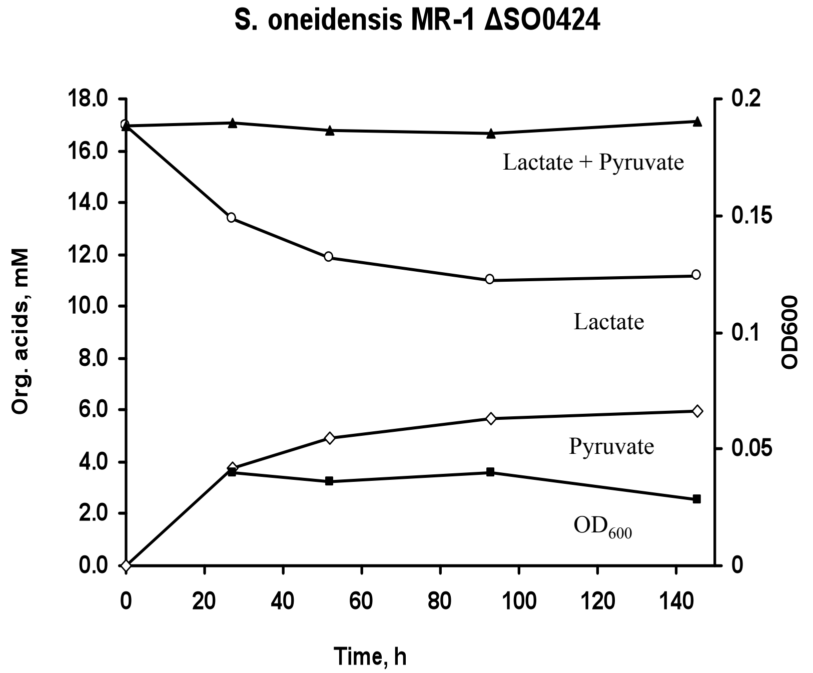

Figure S7. Aerobic growth of *S. oneidensis ΔSO0424* cells in M1 medium supplemented with 18 mM D,L-lactate. 70 ml serum bottles containing 15 ml of medium were used. Periodically 0.7-0.8 ml samples were withdrawn to measure OD_{600} and organic acids in 0.22 μm filtrates of culture.
